# Sub-national health accounts: Experience from Punjab State in India

**DOI:** 10.1371/journal.pone.0208298

**Published:** 2018-12-10

**Authors:** Pankaj Bahuguna, Indranil Mukhopadhyay, Akashdeep Singh Chauhan, Saroj Kumar Rana, Sakthivel Selvaraj, Shankar Prinja

**Affiliations:** 1 School of Public Health, Post Graduate Institute of Medical Education and Research, Chandigarh, India; 2 Public Health Foundation of India, New Delhi, India; BITS Pilani, INDIA

## Abstract

**Introduction:**

Public health spending in India has been traditionally one of the lowest globally. Punjab is one of the states with highest proportion of out-of-pocket expenditures for healthcare in India. We undertook this study to produce the sub-national health accounts (SNHA) for Punjab state in India.

**Methodology:**

We used System of Health Accounts (SHA) 2011 framework for preparing health accounts for Punjab state. Data on health spending by government was obtained from concerned public sector departments both at state and central level. Estimates on Out-of-Pocket Expenditures (OOPE) expenditure were derived from National Sample Survey (NSS) 71^st^ round data, Consumer Expenditure Survey (CES) data and Pharmatrac. Primary surveys were done for assessing health expenditure data by firms and non-governmental organizations. All estimates of healthcare expenditures reported in our paper pertain to 2013–14, and are reported in both Indian National Rupee (INR) and United States Dollar (US $),using average conversion rate of INR 60.50 per US $.

**Results:**

In 2013–14, the current health expenditures (CE) in Punjab was INR 134,680million (US $ 2245 million) which was 4.02% of its gross state domestic product (GSDP).However, public spending on health was 0.95% of GSDP i.e. 21% of the total health expenditure (THE), while 79% was private expenditure. In per capita terms, THE in Punjab was INR 4963 (US $ 82.03). In terms of functions, medical goods (41.6%) and curative care (37%) consumed larger share of expenditure in the Punjab state. Households spent 52% of expenditures for medicines and other pharmaceutical goods. Risk pooling mechanisms are being adopted to a lesser extent in the state.

**Conclusion:**

The healthcare in Punjab is largely financed through private OOPE. Currently, public health spending in Punjab is inadequate to meet the healthcare demands of population, which is less than 1% of state’s GSDP. Monitoring public resources is very important for better resource allocations. Health Accounts production is useful in order to assess future trends and impact of health financing policies on goals of universal health coverage and should be made a part of routine monitoring system both at national and sub-national level.

## Introduction

Central to the discourse on Universal Health Coverage is to ensure financial risk protection which essentially means reorganization of finances to move away from direct out-of-pocket expenditures (OOPE) based financing to various kinds of prepayment based and progressive financing systems. Any systematic assessment of a country’s progress towards UHC perquisites an estimation of how much money is spent on healthcare, how funds are mobilized and allocated into various kinds of health care functions and also identify the sources and reasons of OOPE.

National Health accounts (NHA) as an accounting framework help us to understand the magnitude and pattern of health spending in an economy, and the nature of flow of funds within the health care system. It also facilitates identification of key sources of funding which include government, households, private firms, non-government organizations and their relative importance to total health economy. Further, the state health accounts provide us with an understanding of how much money is spent on various health care commodities such as drugs, diagnostics, hospitals charges, consultation fees, etc. Critical health financing questions, such as the extent of prepayment and risk pooling mechanisms in the state is explicitly captured by health accounts. Many developed and developing countries use NHA data to monitor their progress towards UHC. India has produced NHA twice earlier i.e. in year 2001–02 and 2004–05 and in recent times twice in 2013–14 and 2014–15[1, 2]. Barring an attempt almost 2 decades ago, no comprehensive attempt for mapping sub-national health accounts is available in peer-reviewed literature [3].

Globally, increased public financing of various kinds are being considered the quintessential element in progress towards UHC. Compared to other developing countries and countries in the neighbouring regions, public health spending in India is one of the lowest worldwide[4]. In 2013–14, public health spending share as a percentage of GDP in India was only 1.15% which translates to Indian national rupee (INR) 1,042 per capita at current prices[5, 6]. This is equivalent to United States dollar (US $) 62, which was lower than its neighbours like Sri Lanka (US $93) and Thailand (US $214) or other BRICS nations [5]. In 2014, per capita general government expenditure on health in Brazil, Russia, China and South Africa were US $607, US $985, US $408 and US $554, respectively [5, 7].

In a country like India with a federal structure, health is a State subject with small sub-set of programs coming direct purview of Central government. Hence, State allocations to health are predominant contributor of public spending for health, which also lead to wide inter-state variations. Secondly, there is a fiscal deregulation of taxation wherein states keep a greater share of overall tax. This implies that states have a higher fiscal space to decide health sector allocation. More alongside the fiscal deregulation, Central government is reducing its share of spending on health [8, 9]. Thirdly, there is a renewed focus on decentralized planning for health at state and district level under the National Health Mission (NHM) [10].

All these factors point to a policy need to map health accounts not just at national level, but more importantly at State level. In order to address this evidence gap, we undertook the present study to map the sub-national health accounts of Punjab state in India.

## Health accounts framework, materials and methods

### Ethics statement

The study was approved by institutional ethics committee of Post Graduate Institute of

Medical Education and Research, Chandigarh, India.

### Health accounts framework

The main objectives of a health accounts exercise are to capture how much money is spent on health and identify the sources of resources the financing schemes through which resources are mobilised, the intermediaries through which the funds are routed and the functions performed by the health system and identify the various providers involved service delivery. The system of health accounts (SHA) 2011 framework provides a systematic way to define the boundaries of health care expenditure and classify expenditure incurred by various entities [11]. As per this framework, four components of healthcare system i.e. Governance, resource generation, financing and service delivery are linked to three axis of health accounts (HA) framework i.e. consumption, provision and financing [11].

According to the SHA 2011 framework, expenditure on various kinds of health care activities (functional classification) are considered including health promotion and prevention; diagnosis, treatment, cure and rehabilitation of illness; caring for persons affected by chronic illness, providing community health programs, governance and administration of the health system etc. Health- related activities those are provided as ‘aid’, do not belong to healthcare functions, e.g. provision of long-term social care, enhancing integration of disabled persons, control of food hygiene, drinking water, environmental protection.

Current expenditure on health care considers final consumption expenditure of resident units on health care goods and services during the accounting period. Current expenditure does not include the expenditure on capital (i.e. the total value of the assets that providers of health services have acquired during the accounting period). We used accrual method of accounting and therefore, all resources consumed for healthcare in the state of Punjab in the fiscal year April 2013 to March 2014 (commonly referred as 2013–14), were considered in our study. Besides the resources which were procured directly through the state and central financing, we also considered the resources which were allocated to Punjab state through in-kind supplies by central government and donors.

We added some sub-classifications to SHA 2011 classification which were suited to financing schemes operational in Punjab. At third digit level classification, we created a sub-category for the tax funded government based voluntary scheme (HF.1.1.3). This sub-category include mainly publicly funded health insurance schemes like Rshtriya Swasthiya Bima Yojna (RSBY).

Although the NHA framework used in the manuscript is drawn from global SHA, 2011 framework, the methods used in the manuscript is nationally relevant and specific to states in India, given that a substantial government funding and provision of services is in states’ own domain. The methods used in the manuscript, in specific to households’ OOP, enterprises spending, and non-profit institutions serving households (NPISH) was developed largely by the study team. The study team immensely benefited from several interactions and deliberations among technical teams involving the National Health Systems Resource Centre (NHSRC) and the NHA Expert Group [6]. These consultations contributed towards designing surveys, tools and techniques to validate the survey data to scale up sample estimates to state level, identifying the universe of enterprises & NPISH, in specific to enterprises and NPISH spending. In respect to households’ OOP estimates, Punjab’s health accounts estimates utilise similar methods as adopted at the national level by NHSRC.

### Data collection

Various sources of revenue depicted in the section on health financing flows have various sources of data. The task of Health Accounts is to be able to capture all the various sources appropriately. Table 1 depicts the data from various entities used for generating HA for Punjab and their data sources.

**Table 1:** Data from different entities and their source used for SHA-Punjab, 2013–14.

Entities	Information Source
*State Department of Health and Family Welfare*, *Government of Punjab*	*Detail demand for grants*
*National Health Mission (NHM)*	*Financial Monitoring Report*
*Urban Local Bodies (ULB)*	– *Primary survey of 6 municipal corporations;* – *Directorate Department of Local Government Punjab;* – *Statistical Abstract Punjab 2014*.
*Railways*	– *Demands for grants (DDGs) volume-2 for expenditure of the central government on railways for the year 2015;* – *website of health directorate of Indian Railway*
*Employees’ State Insurance Corporation (ESIC)*	*Annual report*, *2013–14*
*Rashtriya Swashthiya Bima Yojna (RSBY)*	*Claims data*
*Medical Reimbursements (Other Departments)*	– *Detail demand for grants (all departments);* – *Reserve Bank of India (RBI) State finances*
*Private expenditures*: *Out-of-pocket expenditures (OOPE)*	– *National Sample Survey (Morbidity and Health Care Survey*: *NSS 71st round*, *Schedule 25*.*0 collected during January-June 2014;* – *Consumer Expenditure Survey (2011–12);* – *Pharmatrac data of 2014;* – *Population Census (2001 & 2011) and* – *Insurance Regulatory and Development Authority (IRDA) information on Health Insurance 2013–14 has been used*.
*Post Graduate Institute of Medical Education and Research (PGIMER)*	*P*.*G*.*I 47*^*th*^ *annual report 2013–14*
*Private firms*	*Primary survey of firms*
*Non-Governmental Organizations (NGOs)*	*Primary survey of NGOs*

#### Public expenditures

The most important government intermediary related to health is the Department of Health and Family Welfare (DHFW) in the state of Punjab. DHFW mainly manages the finances routed through the state treasury the details of which can be obtained from the detail demand for grants (DDGs) submitted by the line department to the department of finance as their expenditure requirements for coming financial year at the time of preparation of state budget. The DDGs contain audited expenditure incurred two years back. Various other state departments spend considerable amount of money on health. ‘State Finances: a study of budgets’, an annual publication of Reserve Bank of India, which captures public spending by all states, has been used to identify the departments which incur 80% of all the revenue expenditure of the government. A thorough scrutiny of all the DDGs for reference period have been undertaken to identify the health related expenditure of other departments.

Healthcare expenditures incurred under the NHM, a Union government program, was obtained using the Financial Monitoring Reports (FMR) from the State Health Societies (SHS for 2013–14. Several other central government ministries provides health benefits to the people of Punjab. First and foremost is the Post-Graduate Institute of Medical Education and Research (PGIMER), Chandigarh, a premier medical education and research institute. Expenditure incurred by PGIMER has been captured from the 47^th^ annual report of the institute (2013–14) [12]. This report provided information on both plan and non-plan budget of the institute for 2013–14. Another crucial central government intermediary is the Ministry of Railways which provides health care to its employees through a chain of regional hospitals and dispensaries and also run preventive and public health programs. Two secondary data sources i.e. the demands for grants (DDGs) volume-2 for expenditure of the central government on railways for the year 2015 and the website of health directorate of Indian Railway has been used [13]. Expenditure by the Employees’ State Insurance Corporation (ESIC) has been captured using the data available in the annual report of ESIC for the year 2013–14 accessed from its website [14]. It provides state-wise expenditure information under the head of state expenditure, expenditure on Model and ESIC Hospitals and Super Specialty hospitals. Expenditure by RSBY, a government sponsored health insurance scheme, run by Ministry of Labour, was captured through the claims data for the 2013–14, obtained from state government.

Healthcare expenditures data for local bodies was sourced through both primary surveys and secondary data sources like Department of Local government, Punjab and Statistical Abstract 2014 released by Directorate of Economics and Statistics, Punjab[15]. We also undertook primary data collection in 6 major municipal corporations of the state, namely Bathinda, Patiala, Jalandhar, Mohali, Amritsar, and Ludhiana. Lastly, health spending by other departments was obtained from two secondary sources i.e. detail demand for grants documents of all departments and Reserve Bank of India (RBI) State finances study (2013–14) [16].

#### Private expenditures

**Fig 1:**
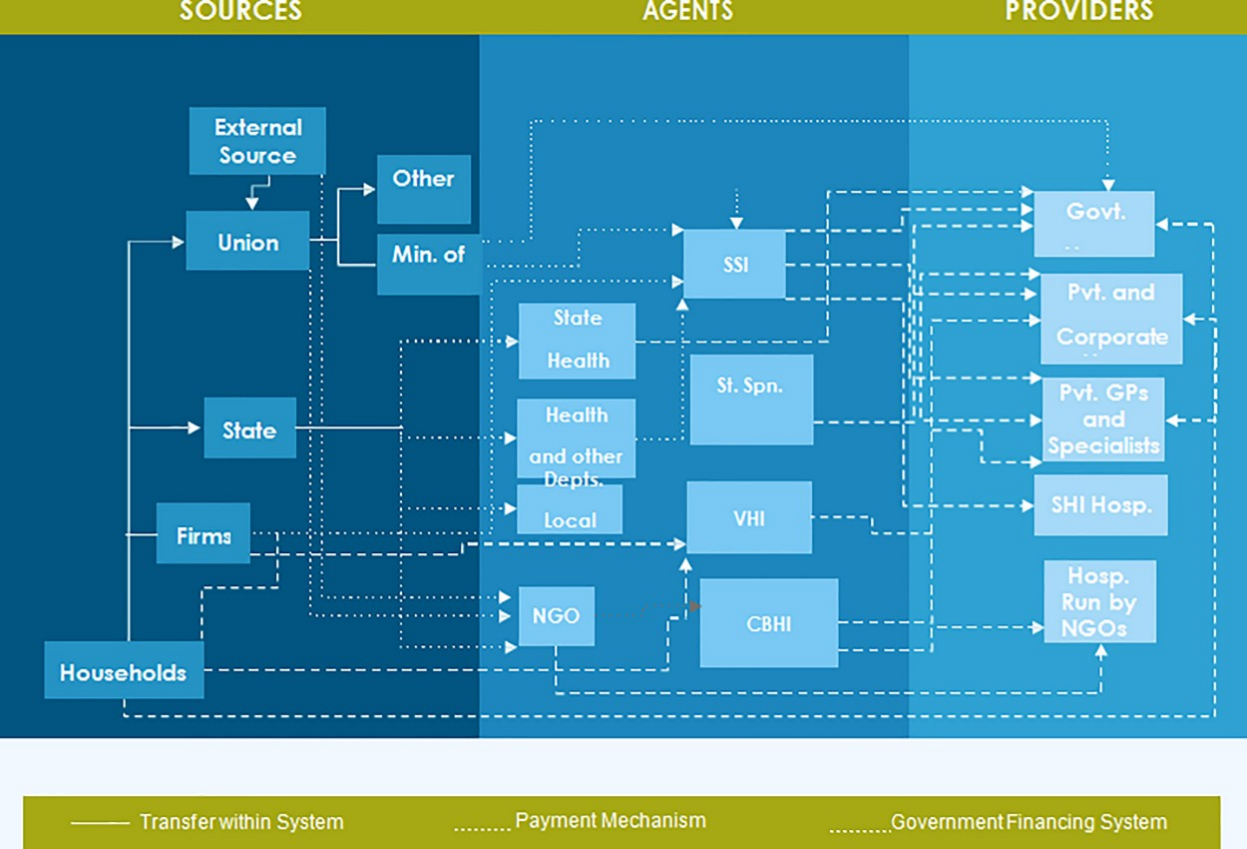
Health financing flow in Punjab.

As depicted in Fig 1, households, firms and NGOs are the private sources of health care funds. Estimates of household expenditure were derived using various data sources. Information on direct OOPE on -patient care, out-patient care, child birth, antenatal care (ANC), postnatal care (PNC) and prepayment in the form of premium for voluntary health insurance (VHI) was derived by analyzing the unit records of National Sample Survey Organisation (NSSO) 71^st^ round survey on Social Consumption related to health [17]. OOPE for healthcare in the NSSO survey is elicited in terms of expenditures incurred for doctor consultation, medicines, diagnostic tests, procedures, travel, boarding, lodging, food etc. This survey was scheduled in 2014 and thus, represents the information for a period which corresponds to our study time period. Expenditure on medical devices and family planning were estimated using the NSSO consumer expenditure survey (CES)[18]. Private expenditure on vaccination is another important component of OOPE in India. We have captured OOPE on vaccination using Pharmatrac data. To capture health spending by enterprises, a nationwide primary survey, with state wise representation of incorporated enterprises was undertaken. This survey covered 32 sample enterprises and all units operating in the state (Details given in supporting information). Majority of enterprises reported spending on ESIC, though the absolute value of this expenditure was not very high. Similarly a primary survey of 148 NGOs in Punjab comprising of all large NGOs and sample of micro/small/medium size to capture health expenditure by NGOs (refer to supporting information for more details).

### Data analysis

Excel spreadsheet was used to enter the data obtained from various sources and intermediaries (interchangeably used with entities) and design the matrix for various set of classifications. Each line item of expenditure was then classified into various financing sources, health care functions and health providers. The classifications were done based on SHA-2011 framework, which were subsequently verified with state government officials. Details about data analysis methodology for specific entities is given in supporting information. Our study estimates of healthcare expenditures pertains to year 2013–14 and are reported in both Indian National Rupee (INR) and United States Dollar (US $) for wider comparability (Tables 2 and 3). Average conversion rate of INR 60.50 per US $ in 2013-14was used[19].

**Table 2:** Key indicators of health expenditure for Punjab during the year 2013–14.

Indicators	INR million	US $ million	As % of GSDP	Per capita (INR)
**Current Public Expenditure (I)**	28800	476	0.86%	1038
**Public Capital Expenditure (II)**	3020	50	0.09%	109
**Total Public Expenditure (A = I +II)**	31820	526	0.95%	1147
**Private Expenditure (B)**	105880	1750	3.16%	3816
**Current expenditure on health (C = I +B)**	134680	2226	4.02%	4854
**Total Health Expenditure (Including current and capital) (D = C+II)**	137700	2276	4.11%	4963

Note: INR = Indian National Rupee, US = United States, GSDP = Gross State Domestic Product

Current expenditure excludes spending on capital. GSDP for Punjab state (2013–14) is INR 3347140million.

**Table 3:** Distribution of current health care expenditure by various schemes in Punjab, 2013–14.

Entities	INR* in million	Share	Per capita
Central Government	4840	3.6	175
State Government	20530	15.2	740
Local bodies	660	0.5	24
Social Insurance	2740	2.0	99
Tax financed Insurance	20	0.02	1
Households	102700	76.3	3702
Firms	2620	2.0	94
Non-profits	560	0.4	20
Total Health	134680	100	4854

*INR = Indian National Rupee

#### Apportioning statistics

Classifying various expenditure items into the SHA classifications is a challenging task, which often require apportioning line items into more than one sub-categories. For instance, funds going to public hospitals through DDGs are divided into inputs, whereas the facility performs various functions using those inputs, which are of interest in SHA exercise. In order to apportion shared expenditures for various functions, we drew cost ratios from recently published literature on costing of public health service delivery undertaken northern states including Punjab [20–27].

The ratio of economic cost for providing health care services to a patient through out-patient (OP) and inpatient (IP) was assumed to be 1:4.96 and 1:3.98 for primary health centre (PHC) and community health centre (CHC) respectively, [22] (Table A in S1 File). In district hospitals, service provisioning is largely of curative nature, the expenditures were apportioned into three main categories i.e. outpatient care, inpatient care and day care services following the SHA 2011 functional classification. Expenditure on day care was assumed to be 10%, using existing literature[28]. After apportioning the day care part, remaining part was apportioned for outpatient and inpatient curative services. The ratio of resources consumed for providing health care service to a patient through OP:IP department was assumed to be 1:4.97 [21] (Table B in S1 File).

A tertiary care hospital (PGIMER) located in Chandigarh (U.T), caters to population of five states including Punjab. As per the hospital statistics by the regional distribution of beneficiaries, Punjab had a share of 30% in the total patient load[29]. Total expenditure of the institute in 2013–14 was apportioned into two main classifications i.e. teaching and patient care. Some part of the overall grant received for the institute is specifically listed under ‘Education and Training’. These expenditures were considered under capital expenditures as per the SHA 2011 definition. Besides this, the salaries/compensations given to the institute faculty, students and administrative staff who are involved directly or indirectly in teaching and training programme was also apportioned based on their time allocation for different activities.

For time allocation estimation, few departments’ were randomly selected and their teaching rosters, schedules of journal clubs, seminars and other teaching/training activities for past 6 months were obtained. Besides the classroom teaching, the time spent on clinical teaching rounds in the inpatient wards and case discussions in outpatient departments were also considered by interviewing faculty members and resident doctors. Apportioned share of teaching was 10.7% of overall salary expenditure and is used as final apportioning factor for teaching.

Secondly, the expenditure share for patient care was further disaggregated into three main classifications which is outpatient care, inpatient care and day care. The ratio of resources consumed for providing health care service to a patient on OP: IP: Day care was assumed to be 1:10:2 using the unit cost estimates from literature [30]. Finally, all the disaggregated expenditures were apportioned for Punjab which was 30% of total expenditures based on its share in terms of patient load (Table C in S1 File).

#### Double counting issues

Data from different sources was utilized for SHA exercise. Simple summation of data from different sources can lead to double counting of expenditures. If we observe the fund flow mechanism, then we can identify that for a state, Union government (i.e. Centre), State government, Local Bodies, and Firms are the source of fund and act as Financing Sources. From the centre, funds flow to the State Health Society, State Departments, and Local Bodies. For example, the expenditure on health by the Local governments is reflected in the Treasury budget in the form of block grants from state to the local bodies; Local government budget in the form of revenue collected from Centre, State and own sources, and Central budget in the form of grants from the centre to the local bodies. In this study, in order to avoid double counting we have deducted the grants that are coming from the state health department, both to rural and urban local bodies. Expenditure of state departments for local bodies is included at the state level but excluded at the local bodies’ level.

Similarly, Enterprises spend on healthcare mainly via three routes: employees’ health benefits, corporate social responsibility and expenditure incurred on health facilities owned by themselves. A proportion of overall enterprises spending is in the form of group insurance to their employees through ESIC. Corporate Social Responsibility (CSR) is a mandatory spending by corporates as per the rule by government of India, under Section 135 of the Companies Act, 2013, and thus, corporations spend a substantial portion of the total CSR amount for promotion of healthcare. They spend it either on running their own NGOs, or by funding to other NGOs. Since we are capturing both ESIC and NGO expenditure separately, we have not taken these two expenditure heads under Enterprise expenditure to avoid double counting. Enterprises’ spending on health through ESIC routes has been estimated directly from the data obtained from official sources.

## Results

### Health financing flows

Health Accounts helps us understand the financial flows with in health system, identify the various sources of money, the intermediaries which are involved in paying the health care providers. Fig 1 gives an overview of the financing flow mechanism of Punjab. Financial flow into health sector in Punjab can be divided into three parts, source of fund for health sector, intermediaries which includes different agents that spend money on health and lastly there are health providers. There are primarily three sources, first it is the money disbursed by the state and central government from the revenue generated from different types of taxes and external aid received by both the governments. Other important source is the out of pocket expenditure that takes place at the household level and lastly there is money spent by the private firms on its employees. Health expenditures through various entities were covered under SNHA Punjab which include centrally sponsored schemes like NHM and Central government Health Schemes (CGHS), PGIMER a tertiary level hospital established by the government of India and financed under central sector scheme, health expenditures by other public sector departments of Punjab in the form of medical reimbursement and employee health insurance, health expenditure of central Railway ministry, State Department of Health, ESIC, Tax financed insurances like RSBY, Department of Local Bodies (Urban Local Bodies (ULB) and Rural Local Bodies (RLB)), Private firms and households. Detailed text is available in supporting information.

Goodness and fairness are two critical aspects while evaluating a health care financing system. Goodness would mean that the system would be able to mobilise adequate resources to meet most of the healthcare needs of the population. Goodness can be measured by the share of resources of the economy that are devoted towards healthcare. Fairness of health financing system is measured by effective reduction of OOPE and a corresponding expansion of prepayment based systems, which pool financeable health risks effectively.

In 2013–14, the current health expenditures (CHE) in Punjab was INR 134,680million (US $2,245 million) which was 4.02% of its Gross State Domestic Product.When capital expenditure is included with CHE, we arrive at total health expenditure (THE), which was INR 137,700million (US $2,276 million)around 4.11% of GSDP. In per capita terms, THE is INR 4,963 (US $82.03). Total public expenditure including capital is INR 31,820million (US $525.9 million), equivalent to 0.95% of GSDP (Table 2). General government Expenditure (GGE) constituted 21% of the total health expenditure (THE), while the remaining 79% was various kinds of private expenditure.

### How are the health care resources mobilized?

**Fig 2:**
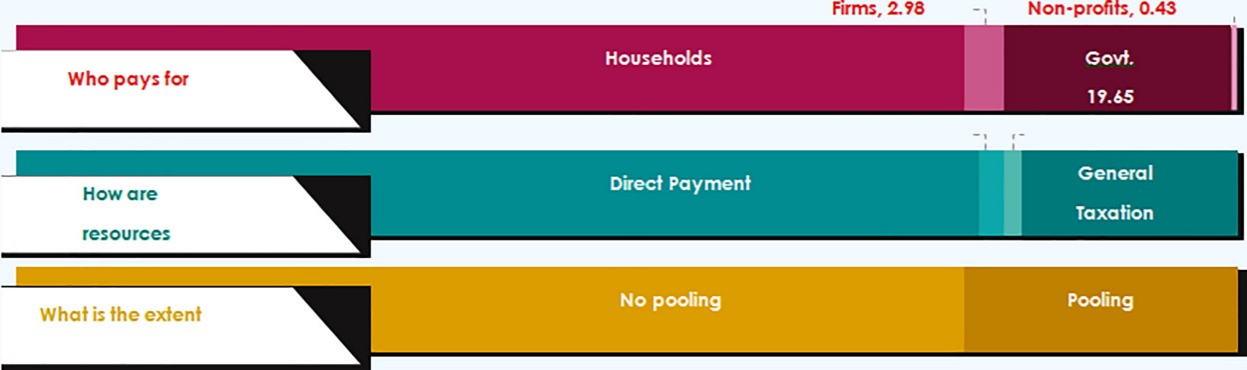
Various financing dimensions in Punjab, India.

Core health financing function is to mobilize resources in a manner that helps pool health care risks and allows cross-subsidization. Popular methods of resource mobilization for health care are general government revenues, including taxes; mandatory contributions from employers and employees for social insurance, voluntary contributions from employers for provision of health care or financial protection and contributions from various domestic and international not for profit organizations and through these forms reduce the role of direct OOPE. What we observe in India is an overwhelmingly high dependence on direct out of pocket payments for health care (76.3%), while a small part of direct payments are made by firms for their workers. Only 19.4% of resources are mobilized through taxes and other forms of government revenue; whereas only 2% resources are mobilized through SHI and only 1.6% of resources are mobilized through VHI (Fig 2).

### Who pays for health care in Punjab?

As we try to track the sources of health financing resources, we observe households pay for more than three quarter of all health care expense in the state (76.64%), whereas general government expenditure (GGE) contribution is less than a fifth (19.98%) (Fig 2). Firms and NGOs taken together contribute to only 3.4% of total spending i.e. CHE.

As we break up general government expenditure by various levels of government, we observe that state government, comprising of Department of Health and other line departments finances the major part of the government expenditure and more than a seventh of CHE (15.2%). Financing by other departments (supporting information) was mainly consumed for medical reimbursements of employees. Central government spending is routed through various channels including the NHM, central government funded tertiary care institutions like the PGIMER, Chandigarh; the Railways, in-kind supplies of vaccines, medicines for programs, family planning material from centre to states. All the various central government sources together constituting 3.6% of CHE. The Employees' State Insurance Scheme caters to 2% of CHE. Contribution of local bodies is limited to 0.49% of CHE (Table 3).

### Which health care services are financed?

**Fig 3:**
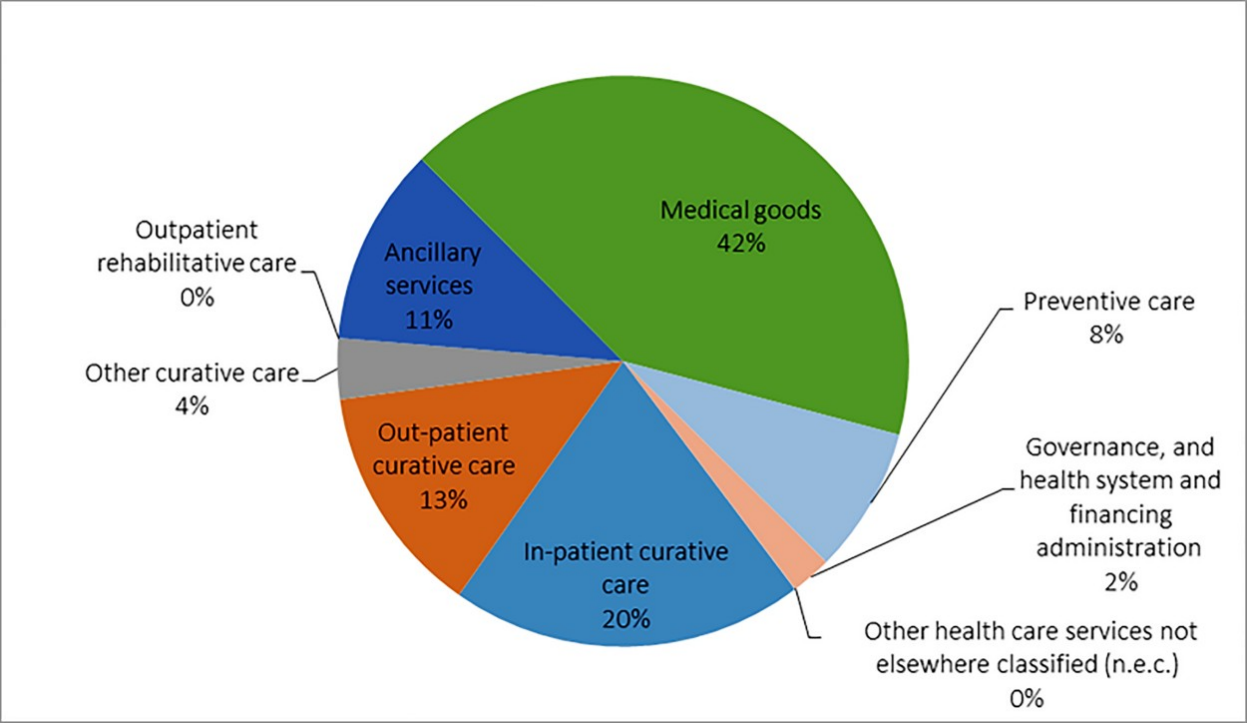
Distribution of healthcare expenditure by functions in Punjab, 2013–14.

To understand the priority accorded to various health care functions and how these functions are financed provide is important policy insights including possible reprioritization of finances. Among the various functions expenditure on medical goods had the highest share (41.6%) in the state (Fig 3). These include mainly spending on medicine and other medical consumables which are not provided by the public or private health system and households purchase themselves often without prescription. Around 37% of the health care resources were spent to fund curative care which include 20% spent on IP care, whereas 13% are spent on OP care. Ancillary services like ambulances and diagnostics tests are other important function with a share of 11% in the CE (Fig 3). It has to be noted that expenditure on curative services does not include expenditure on medical goods and diagnostics which are obtained directly by households. A major part of OP are in the form of the medical goods and diagnostics procured directly.

**Fig 4:**
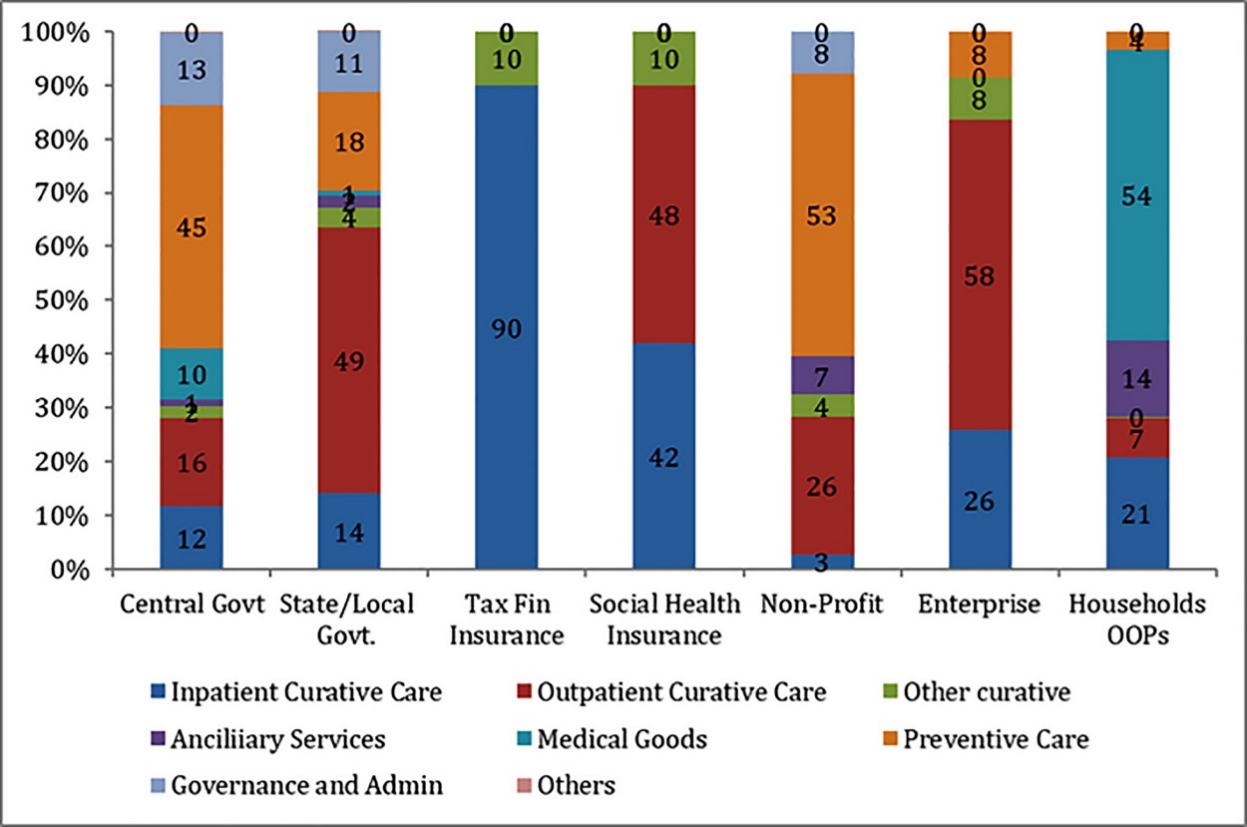
Distribution of type of healthcare provided by financing scheme in Punjab, 2013–14.

Out of the total public expenditure, maximum spending of around 60.8% was on curative care of which outpatient curative care comprised of31.4% (Fig 4). Expenditure on preventive care recorded the second highest spending of 24.2%, out of which information, education and counselling programmes had a major share of around 56%. Spending on governance, financing and administration consumed around one-tenth of the overall public health spending. Expenditure towards medical goods (2.5%) and ancillary care services (1.8%) was on the lowest (Fig 4).

### What kind of care is purchased by households?

Out of the total private expenditure, maximum spending i.e. 52% was on pharmaceuticals and other medical non-durable goods. Expenditure on the curative care was 30% of the total private spending, constituting second highest expenditure. Of the total spending on curative care, inpatient curative spending had a major component of 70%. Spending on ancillary services and preventive care was about 14% and 4% of the total private expenditure on health care respectively (Fig 4). As noted earlier a major part of the OP care is in the form of medical goods and diagnostics and if we add all these components major part of the OOPE is in the form of OP.

### Who provides health care?

**Fig 5:**
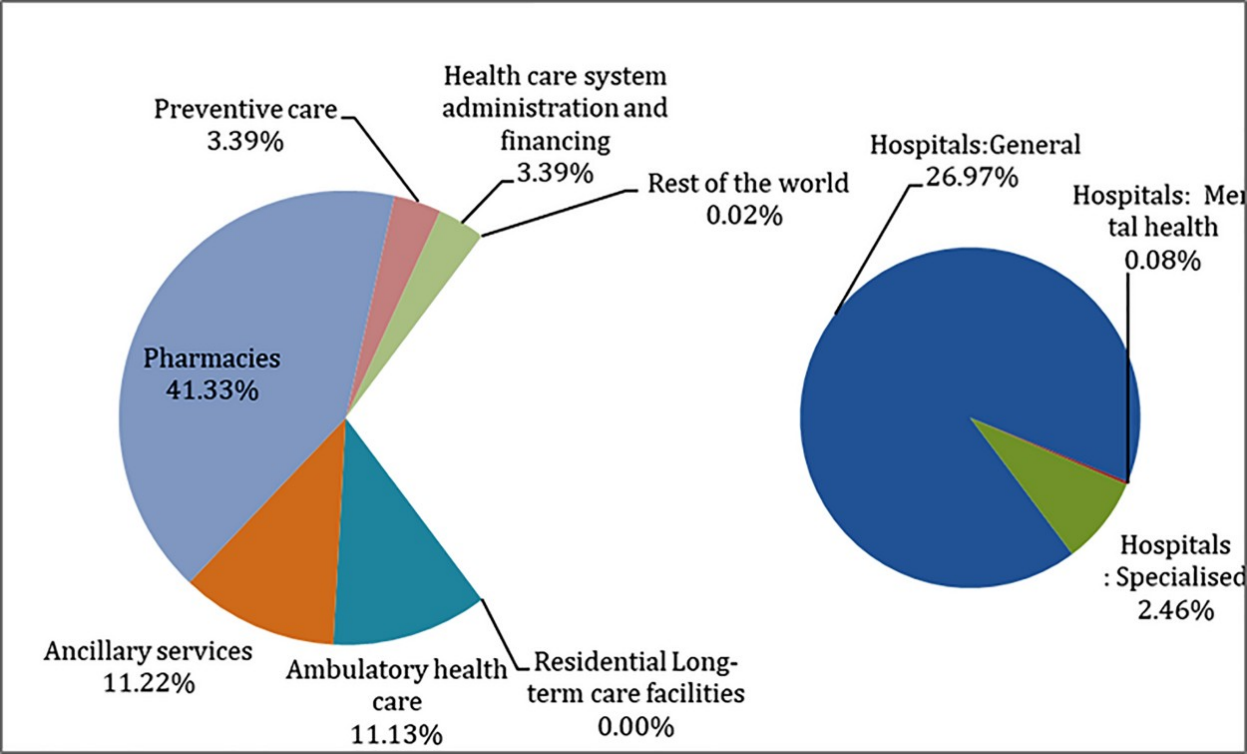
Distribution of health care expenditure by providers in Punjab, 2013–14.

The classification of health care providers serves the purpose of identifying various organisations that contribute to the provision of health care goods and services and classify provider units into common, internationally applicable categories described by SHA 2011 (SHA 2011 pp 122). In Punjab, maximum spending (41.3%) was on the providers of medical goods, with pharmacies consuming almost all the share (Fig 5). Expenditure on providers of hospitals (29.5%) especially general hospitals (27%) recorded second highest share. Expenditure on providers of ambulatory and ancillary services was almost similar, with each consuming around 11% of the total spending. Further, health care system administration and financing had a share of around 3.4%. Health care spending on providers of preventive care also had a similar limited role to play with their share hovering around 3.39% (Fig 5).

### What are the various inputs used for producing health care services in Punjab?

Factors of provision are the valued inputs that are used in the process of production of health care. Provision involves a mix of factors of production–labor, capital and materials and external services to provide health care goods and services. It refers not only to health-specific resources but also to the non-health specific inputs needed to generate health services.

In terms of factors of provision, maximum amount (86%) of overall public health expenditure was spent on the wages and salaries of human resource. Rest of the expenditure is divided under several heads such as health goods (4%), non-health goods (1%), health services (5%) and non-health services (0.42%).

In case of private spending maximum amount was spent on the consumption of medical goods (54%) and medical services (including diagnostics) (32%) followed by expenditure on non- health services (3.7%).Expenditure on the wages and salaries of the employees consumed around 10% of the total private health care expenditure.

## Discussion

The paper, based on the Sub-National Health Accounts for Punjab, has attempted to address some key questions related to health care financing making use of the evidence related to health care financing in Punjab. Our attempt to capture total resources devoted to health shows that in 2013–14, THE in Punjab was INR 137.7 billion (US $ 2276 million), which is 4.11% of state’s GDP. In per capita terms, THE was INR 4963 (US $ 82.03). Overall resources mobilised for health is slightly higher than the national average of 4.02%, but lower than countries with similar levels of per capita income [7]. The main reason for low overall resource mobilisation could be found in lower public spending- less than a fourth of THE. It has to be noted however that compared to 2001 (17%) and 2005 (18%), the years for which we have comparable numbers, there is some increment in public spending over the years. [1, 2]. Share of public health expenditure was again less than one-fourth (23%) of THE, with OOPE contributing 76.6%. Per capita health spending by government in the state was INR 1147 (US $18.96). Only around one-fourth (24%) of the health expenditures were routed through risk pooling mechanisms. Major driver of service provisioning was curative care which accounted for three-fourth of current health expenditures.

General government revenue based financing remain the mainstay of financing health in developing countries, particularly among those who have been able to provide better financial protection. As evident from our study limited expansion of general government revenue based financing and other low penetration of various forms of social insurance or voluntary insurance mechanisms, households essentially depend on the direct OOP based financing to meet their health care needs. Expansion of social insurance mechanism like the ESIC remains a challenge because of the structural reasons, as large part of the economy is in the unorganized sector.

We noticed that in 2013–14, OOPE burden was more (76.3%) in Punjab compared to national level i.e. 69.1%[6]. In Punjab households’ expenditures on healthcare has increased in last 15 years. It was 55% in 2001–02 which increased to 76.3% in 2013–14 [3]. At national level, public share in total health care expenditures has improved from almost 20% in 2001–02 and 2004–05 to 28.6% in 2013–14, while in Punjab the rate of improvement has been poor i.e. 17% in 2001–02, 18% in 2004–05 to 21.4% in 2013–14 [1, 2, 6]. This indicates that in the period from 2001–02 to 2013–14, though the government spending on healthcare has increased in absolute terms but still remains insufficient to cater the needs of the population.

A direct OOP based system is regressive and leads to inequities in terms of health care utilization, health care financing and financial risk protection [31, 32]. Not only does it have considerable equity implications, it brings in several kinds of technical and allocative inefficiencies. Pharmacies as a healthcare provider consumes the predominant share of healthcare expenditures both in Punjab (41%) and India (36%) [6]- When medicines are purchased from retail outlets it increases considerable burden on households in comparison to provision through the system. When large health systems procure medicines in bulk it cuts costs by using monopsonistic power as well as reduces the use of intermediaries in this process. In case of households expenditures, almost half (52%) was spent on purchasing medicines and other pharmaceutical goods. Availability of medicines in public health facilities in Punjab is low [17, 33] which imposes a burden on population utilizing public health facilities to buy medicines form private pharmacies and thus expose them to catastrophic health expenditures and impoverishment [32, 34, 35]. It further causes, private pharmacies in the state to be predominant providers of healthcare (41.33%).

Another facet of healthcare expenditure in India is high spending for curative care service (80%), with merely one-tenth being spent on preventive services which is much lower than the policy prescription [4, 6]. As per the recommendation of Indian national health policy (NHP) document (2017), more than two-third of the resources should be allocated for primary care [4]. Punjab spent less on preventive care (8%) compared to India (9.6%). Health system in developing country like India cannot afford to depend heavily on curative care, particularly strategies premised upon promotion of hospital care and in this process ignore preventive and primary care. Globally, the most effective ways of expanding preventive and primary care is through general government revue financed and directly provided services, even if there is considerable presence of social insurance. The ability of current strategies to expand various kinds of publicly financed health insurance in India remains limited in their efficacy in reducing catastrophic payments until unless these are complemented with effective public provisioning. The strengthening of primary health care has been recommended as a cornerstone of UHC strategy for the north Indian states, including Punjab [36].

### Strengths

We identify few strengths of our study. Firstly, we used the standard methodology of SHA 2011 framework to produce SNHA for Punjab which gives it a wider horizon of comparability both between Indian states and other parts of the world. Second, our study estimates comes at a time when there is a renewed focus on achieving universal health coverage both at national and state level [37] and thus, can provide evidence for redesigning resource allocation. Third, design of health system is usually very complex which involves flows of funds from multiple directions. We did a careful mapping of the possible flows which avoids any double counting. Lastly, top down approach leads to several shared expenditures across different entities. While previous studies have relied on expert opinion, we used primary data from local costing studies [20–24]to determine apportioning statistics. Our apportioning statistics, reported in the study can be useful for future sub national or national HA in India.

### Limitations

Firstly, the SHA-2011 (and previous SHA’s) was primarily designed for organization for economic co-operation and development (OECD) countries in view of several variations in the funding mechanisms in local context, we had to adapt the classification system. National Health Accounts Technical Secretariat (NHATS) did some methodological refinements in SHA framework required in Indian context. Despite this, there exist several unresolved issues [6] like including healthcare expenditures by Member of Parliament (MP) in their constituencies through MPLADS (Members of Parliament Local Area Development Scheme) funds, non-government funding to autonomous institutes etc. Henceforth, more research or expert consultation is recommended to make SHA framework more suitable for India and states. Second, we could not obtain data on healthcare expenditures by Ministry of Defense due to their rigid data sharing polices. Third, we did not undertake disease-specific HA and public resource tracking activities as a part of this study which is an important aspect of any HA. Lastly, in view of decentralized planning in India, in future, HA should be undertaken at district level too.

### Conclusions & policy implications

Global experience suggest that any move towards UHC pre-requisites an expansion of prepayment based systems, where financeable health care risks are effectively pooled. In a country where there are structural limits to expansion of social insurance, it is imperative that expansion of prepayment and risk pooling is done essentially through increased public spending. Public health spending in Punjab is inadequate to meet the healthcare demands of population, with a share of mere 23% in THE, which is less than 1% of state’s GSDP. As proposed by the National Health Policy 2017 and many other policy documents which precede, public spending on health should be raised at least to 2.5% of GDP [4]. Secondly, we found that preventive and promotive services in state have failed to attract the health financing with just 8% being spent on these components. With inadequate public financing and risk pooling mechanisms in the state, population is bound to make direct payments for purchasing healthcare, particularly for medicines. It results in CHE and impoverishment among the vulnerable population subgroups mainly accessing the unregulated private healthcare sector [32, 38]. Fifth, monitoring public resources is very important in view of reforming resource allocations for producing better health for money. Government should consider increasing their level of spending encouraging the public health services and improve availability of medicines in public facilities. Provisioning of free medicines in public facilities not only becomes effective in providing financial protection, these play crucial role in improving efficiency of health systems [39].

Currently, HA is a not a routine activity in India, so it should be made a part of routine monitoring system both at national and sub-national level. NHATS as technical unit of Government of India has been assigned the responsibility of generating NHA, capacity building and institutionalizing it across Indian states but till these processes gets streamlined, governments should fund for more sensitization and capacity building in this regard. Since health is a State subject in India, major part of public sector financing is the responsibility of the State Governments. Moreover, in view of the decentralized health planning which is emphasized in various policy documents, it is imperative to generate the health financing data at the state level. This will facilitate evidence based health financing policies at State level. The generation of a regular SHA becomes even more important in view of the Government of India’s recent introduction of the strategies for strengthening of primary health care by creation of health and wellness centres; as well as introduction of the Prime Minister’s *Jan Aroyga Yojana* (PMJAY) [40]. Lastly, in future, disease-specific HA and public resource tracking should also be undertaken as a part of HA exercise. Moreover, in view of decentralized planning in India, it is advisable to generate HA at district level too.

## Supporting information

S1 File Supplementary appendix. (DOCX)
