# Supplementary material for: Sub-national health accounts: Experience from Punjab State in India
Source: PLoS One. 2018 Dec 10;13(12):e0208298. doi: 10.1371/journal.pone.0208298 (PMC6287852; doi:10.1371/journal.pone.0208298)
Supplement: S1 File — (DOCX) [file pone.0208298.s001.docx]

**Health Financing Flows**

***Intermediaries***

Intermediaries are the functionaries which receive the funds from the various sources and then channel it further to the providers. The first five entities being government bodies which further channelize funds to NGO’s or for various insurance schemes.

***Financial flow***

Financial flow into health sector in Punjab can be divided into three parts, source of fund for health sector, intermediaries which includes different agents that spend money on health and lastly there are health providers. There are primarily three sources, firstly it is the money disbursed by the state and centre government from the revenue generated from different types of taxes and external aid received by both the governments. Other important source is the out of pocket expenditure that takes place at the household level and lastly there are money spent by the private firms on its employees.

***Centrally sponsored schemes***

Out of money disbursed from the public source which includes both centre and state government the central government contributes mostly in the form of centrally sponsored schemes related to health and other important component is the national health mission (NHM) through state health society at the state level. In 2013-14 there was expenditure under national rural health mission (NRHM) in rural areas and national urban health mission in urban areas (NUHM) as two components of NHM. Apart from these two components centre also spends on its employee in the form of central government health scheme (CGHS).

***Central Sector Scheme***

Fund flow under central sector scheme takes place directly from centre to a particular institute in the state bypassing the state treasury route. One of the crucial Central Sector Schemes in the state is the Post Graduate Institute of Medical Education and Research (PGIMER), an autonomous tertiary level hospital, education and research centre, established by the Government of India as an advanced centre for medical specialties.

***Other State Departments***

Various other departments also spend on health in Punjab. Medical reimbursement, health insurance by certain department and measures for occupational health of its employee are some of the commonly followed practices by other departments.

***Railway***

Expenditure on railway employees by ministry of railways government of India is another central sector scheme operating in the state.

***State Department of Health and Other Departments***

At the state level there is state department of health and also other departments that spends on health. Under health department, expenditure takes place under two broad heads of medical, and public health and family welfare. Various other departments also spent on health related issues in Punjab. Medical reimbursement, health insurance by certain department and measures for occupational health of its employee are some of the commonly followed practices by other departments. Apart from medical reimbursement other departments also spends on different health functions meant for the poor and destitutes.

***Employee State Insurance Corporation (ESIC)***

Employee state insurance scheme provides economic protection to workers and their dependent covered under the scheme. The sources of funds for ESIC department are three different contributors, namely; (i) employees (ii) employers and (iii) state government. Employee’s contribution rate is 1.75% of the wages and that of employer’s is 4.75% of the wages paid/payable in respect of employees in every wage period. Apart from these two contributors, state government also contribute a significant amount of money to the ESIC to run its own hospitals to provide healthcare to its ailing employees. Hence, as far as agents (intermediaries) of health spending are concerned ESIC is the sole agent whereas, ESI hospitals and dispensaries along with private and public empanelled hospitals are the provider of health care.

***Tax Financed Health Insurance Schemes***

To provide financial security against high medical expenses, the Union Government has launched a Health Insurance Scheme, the Rashtriya Swasthya Bima Yojana (RSBY), for BPL households (as defined by the Planning Commission, India). This scheme covers for a maximum amount of INR 30,000 for a family annually. For this scheme, 75 percent of the premium (not exceeding INR 565) will be provided by the Union Government and the rest 25 percent will be borne by the State Government. Under this scheme, the cost of a Smart Card for each family is decided INR 60 per card and beneficiaries have to pay an annual registration charge of INR 30 per family (which is part of the insurance premium to be paid to the insurance provider).

***Local Bodies***

There are two types of local bodies in Punjab: Panchayat Raj institute (PRI) in rural areas and municipalities in urban areas. Urban local bodies (ULB) are of two types Municipal Corporation and municipalities whereas in rural areas it is the gram panchayat working at the village level. Total revenue of the local bodies comprises own resources, grants from the state and central government and loans. Local bodies in Punjab are both source as well as agent in overall fund flow for health. It uses its own revenue sources to spend on health it also channelize money coming from central or state government money to be spent on health. Local bodies spend money range of health care activities which ranges from basic preventive services to curative services.

***Firms***

Enterprises spend on health care through three routes, namely employees’ health benefits, corporate social responsibility and expenditure incurred on health facilities owned by the enterprises.

***Non-Governmental Organizations***

The non-governmental organizations (NGOs) plays role in health system comprise of (a) provider of healthcare services through own facilities, (b) financing agent to manage several healthcare schemes, and (c) run own health financing schemes. There were about ten health oriented projects in recent past by the central Ministry of Health and Family Welfare, where NGOs in Punjab have been actively taken part as health service provider to financing agent (fund management) based on their level of capacity (for instance, Mother-NGO Scheme). All the NGO-schemes are now under provision of the flexi pools in National Health Mission of Punjab. Besides, some NGOs (especially the national counterpart of global NGOs and faith-based organizations) might have their own health financing schemes. As a result of such heterogeneity and plurality in non-governmental sector, the extent of functioning is also varied across provider to financing in the healthcare system. Within this wide spectrum, there are also some providers as rural community based organizations that offer health services at the primary level, and the other end is public trust hospitals.

***Households***

Households spend on health in the form of out of pocket (OOP) expenditure on health and prepayment in the form premium paid to the insurance. Household expenditure is general in the form of direct payment of fees for services. To a limited extent, households also spend money for purchasing private voluntary insurance. Out of pocket expenditure includes use of both inpatient and outpatient services from all kinds of medical services. OOP expenditure includes payment made for health workers for curative services, medicine and preventive services related to maternal and child health OOP is seen as necessarily regressive form of financing and depicts lack of financial protection.

**Data Collection**

***Private expenditures***

**Firms**

Health expenditure in India is financed through various sources. Enterprises’ contribution is one of the components of total health expenditure. This study is being conducted to estimate the contribution of enterprises towards overall health expenditure. These estimates would be used for the wider objective of preparation of national health accounts as well as health accounts for major states of India. Typically enterprises spend on health care through three routes, namely employees’ health benefits, corporate social responsibility and expenditure incurred on health facilities owned by the enterprises. This particular study attempts to capture the expenditure made in these three ways. It attempts to capture the ways in which the expenditure is made within these three categories.

**Sampling and Survey:**

Two separate frames have been used for public sector units and private enterprises. List of PSUs was obtained from Department of Public Sector Enterprises (DPE), Government of India in case of central PSUs and state audited reports from Comptroller General Audit, Government of India in case of state PSUs. Prowess data set from CMIE has been used as frame for private enterprises.

Having determined the sample size for private enterprises, sample has been further distributed proportionally among primary, secondary and tertiary sectors within Punjab. Under primary survey 32 enterprises were covered, having 92 branch units located in Punjab.

PSU sample units were selected based on distribution of public sector units across Punjab. Some adjustments were made to ensure representation of all these states in the PSU sample.

The non-governmental organizations (NGOs) are called by various names, such as not-for-profit institutions, third sector organizations, voluntary organizations, community-based organizations, charitable organizations and so on. The key roles played by this sector in health system comprise (a) provider of healthcare services through own facilities, (b) financing agent to manage several healthcare schemes, and (c) run own health financing schemes. There were about ten health oriented projects in recent past by the central Ministry of Health and Family Welfare, where NGOs in Punjab have been actively taken part as health service provider to financing agent (fund management) based on their level of capacity (for instance, Mother-NGO Scheme). All the NGO-schemes are now under provision of the flexi pools in National Health Mission of Punjab. Besides, some NGOs (especially the national counterpart of global NGOs and faith-based organizations) might have their own health financing schemes. As a result of such heterogeneity and plurality in non-governmental sector, the extent of functioning is also varied across provider to financing in the healthcare system. Within this wide spectrum, there are also some providers as rural community based organizations that offer health services at the primary level, and the other end is public trust hospitals. So, an in-depth statistical information is required to observe the different roles of NGOs in Punjab.

However, in line with the international efforts to improve economic statistics, the National Accounts Division of Central Statistics Office, Ministry of Statistics & Programme Implementation, Government of India had prepared a satellite account to estimate role of NGOs in the economy during 2007-08. This was perhaps an innovative and comprehensive study specially designed for the non-governmental sector through census approach with a special emphasis on those organizations serving the households (called them in national accounting framework as non-profit institutions serving households, NPISH). Comparatively, a recent in-depth statistical information on non-profit institutions in general may well be found by analyzing the official survey on Unincorporated Non-agricultural Enterprises, 2010-11 (67th Round NSSO Data, Ministry of Statistics & Programme Implementation, Government of India). But, this survey covered only 234 sample-NGOs in health sector out of their estimated population around 17000 in India (on an average just about 7 samples per state), and hence hardly effective for reliable estimate of national or provincial levels health expenditure by NGOs. So, a general survey of NGOs was conducted for estimating their health expenditure (see Methodology section for scope, coverage and estimation procedure of the study).

**Non-governmental Organizations**

The estimated health expenditure by NGOs in Punjab is based on national level survey. The health expenditure of non-governmental sector is intended to estimate across all major States and Union Territories through a sample survey of registered NPISH (NGOs serving households). Some important issues related to concept, coverage, sample design and estimation are as follows:

1. In line with the national accounting framework of India, a non-governmental unit is defined as the legal entity which is (i) an organization and (ii) not allowed to distribute any profit, by law or custom, to those who own or control it, and (iii) institutionally separate from government, (iv) self-governing and (v) non-compulsory (CSO, Government of India). The NGOs may operate in different sectors like education, health, recreation, religious, culture and so on in mutually non-exclusive ways, and they may be classified by the purpose they are envisaging like governments, corporates and households. However, the scope of this study is to cover those serving the households but may function differentially in health sector.

2. By considering the operational sector and envisaging the serving purpose, samples are drawn from three mutually exclusive groups: first group is *primary-health NGOs*, a set of NGOs exclusively/ primarily involved in health sector activities, and they are the prime interest in this study. Second group is *subsidiary-health NGOs*, those operated in health sector activities as auxiliary basis. Rest are the third group, *non-health NGOs*, which may occasionally be spent on health as their activity or provide health benefits to their own employees, and hence getting less importance in this study. We are considering only a selected part of non-health NGOs that are fairly bigger in size**^^[[1]](#footnote-1)^^**.

3. The survey questionnaire is especially designed to capture health expenditure of NGOs in terms of different roles they played in health sector as provider of healthcare services, agent for mobilizing funds in health system and primary source of revenue for healthcare financing schemes. There are six broad blocks in the survey schedule comprising identification particulars, basic information with detailed health activities, geographical coverage with employment size, receipts as non-grant and grant with details of health grants, expenditures on all possible heads of health activities, and some basic details on each healthcare facilities, if applicable. The health expenditure of NGOs is estimated by covering all available current account expenditures on health activities except for the medical education and research part. To avoid the double counting, inter-NGOs fund flows have also been excluded. All the identified variables of health activities are finally classified for constructing the matrices of health accounts in SHA (2011) Framework.

4. For survey design, the study is not restricted to any particular official lists of NGOs because none of them are updated recently and/or relevant information for stratification are not readily available in public domain. However, the Planning Commission list of signed up organizations in NGO Partnership System and Directory information of CSO-NAD NPI Census 2007-08 are consulted, and an approach of snowballing network is employed to trace the relevant NGOs. The study follows a multi-stage stratified random sampling technique so that samples from different types of NGOs may assure to be represented in the sample. Given the fact about non-availability of auxiliary information for stratifications except for CSO-NAD NPI Census, the same is used to allocate samples in different levels of strata. Since the list of NGOs are fairly old in the CSO information and hence some NGOs may be stopped functioning and/or new NGOs may be appeared over the period, a state level listing of traced NGOs with relevant stratification variables is being prepared by telephonic communications and snowballing networks, and the same is used to draw samples at different levels of strata. Nevertheless, we are relied on relevant estimates from the CSO information for stratification with the assumption that though the absolute values of different stratification variables may well be changed over time but their rates and ratios in particular state are not changed to a large extent.

5. The CSO Census of NPIs contains all required information like directory of NGOs across states/ primary and secondary areas of operation/ serving institutions/ size of employment, etc. As per the survey design, samples are drawn at all-India level for each group and distributed across nineteen major states as per the concentration of NGOs: Andhra Pradesh including Telangana, Assam, Bihar, Chhattisgarh, Gujarat, Haryana, Karnataka, Kerala, Madhya Pradesh, Maharashtra, Manipur, Odisha, Punjab, Rajasthan, Tamil Nadu, Uttarakhand, Uttar Pradesh, West Bengal and Delhi. The estimates of Punjab are drawn from this national level survey.

6. Sample size is determined for each group separately as per the importance of study. Since primary-health NGOs are expected to play the key role from provider of healthcare services to primary source of healthcare revenues, a more intensive sample plan is designed; the study covers all large primary-health NGOs and samples are drawn at 3% margin of error in 95% confident interval from the rest primary-health NGOs comprising micro/small/medium size, and then distributed proportionally at different stages of stratification (across states, and then size-based strata). A moderate level of samples are drawn (5% margin of error in 95% confident interval) for the subsidiary-health NGOs and distributed accordingly. Since the large and medium NGOs are merely considered for non-health NGOs, samples are drawn at 10% margin of error in 95% confident interval for them. Around 1800 samples are drawn for the survey at national level and the corresponding number for Punjab is 63 (covers 148 individual units of NGOs in Punjab).

7. While the samples in each stratum are drawn randomly without replacement, we are ensuring at least thirty samples in each selected states and a minimum quota of two samples in each size based stratum for standard statistical analysis. Further, a particular stratum may be absent in the samples because none of the NGOs are operating in such stratum (no population). Finally, if any non-response arises, the replacement is allowed with similar characteristics.

**Data Analysis**

***Other departments***

To estimate the health expenditure which comes under the boundary of health care defined by the SHA 2011, we have considered the expenditure from other departments of the state government. From the detailed demand for grants of each of these department sub-minor heads have been identified which are being spent on health items. Boundary considerations have been kept in mind in selecting the sub-minor heads.

Medical Reimbursement

Reimbursement provided to the officials in different departments forms a significant amount of expenditure on health by state governments. Hence taking into account of them is essential while estimating state health account (SHA). However, it’s very challenging to find out the expenditure incurred on reimbursement from each departments’ budget documents considering the fact that there are many departments. Hence, we would use the following methodology to estimate total magnitude of medical reimbursement for the state.

Estimation Methodology

Step-1: Identifying around top 10 departments (or may be more) which cover more than eighty percent of total revenue expenditure of all the departments excluding department of health and family welfare.

- To get the Revenue expenditure data, we have to visit RBI handbooks of statistics on state finance
- To rank departments based on total revenue expenditure and identify top ten department or departments covering at least eighty percent of total expenditure, whichever is more.
- To correspond major heads to demand number and departments.
- To collect DDGs of those chosen departments

Step-2:

- To identify medical reimbursement expenditure from DDGs of those departments and sum up total medical expenditure (TR1= Sum (MRi)); i=1…10)

Step-3: To get the average percentage of medical reimbursement expenditure of those 10 major departments

- To get share of medical reimbursement (MR) to total revenue expenditure (TRE) of each department

$$Ri=\frac{MRi}{TREi} \times100 ( i=1\ldots\ldots\ldots10)$$

- To get the average of share of MR of all 10 major departments

$$R=\frac{\sum_{i=1}^{10} Ri}{10}$$

Step-4: To get reimbursements expenditure for all the departments

- Multiplying the figure (R) into total revenue expenditure of rest of the departments for in the state government

TR2= $R\times TRE$

Total Reimbursement: TR1+TR2

Following the above stated steps, the departments selected for Punjab are

1. Other Fiscal Services
2. District Administration
3. Other Social Services
4. Police
5. General Education
6. Technical Education
7. Sports and Youth Service
8. Art and Culture
9. Other Scientific Research
10. Ecology and Environment
11. Water Supply
12. Labour and Employment
13. Welfare of Scheduled Castes /Scheduled Tribes / Other Backward Classes and Minorities
14. Crop Husbandry
15. Soil and Water Conservation
16. Agricultural Research and Education
17. Other Agricultural Programmes
18. Hill Areas
19. Minor Irrigation
20. Command Area Development
21. Nutrition
22. Food, Storage and Warehousing
23. Civil Supplies
24. Special Programmes For Rural Development
25. Rural Employment
26. Other Rural Development Programmes
27. Civil Aviation
28. Road Transport
29. Inland Water Transport
30. Other Transport Services
31. Social Security and Welfare

Expenditure items already captured through the other department expenditure are not included in the calculation of medical reimbursement.

***Employee State Insurance Scheme (ESIC)***

For ESI we have used the secondary level data available in the annual report of Employee State Insurance Corporation for the year 2013-14 accessed from its website [[1](#_ENREF_1)]. It provides state-wise expenditure information under the head of state expenditure, expenditure on Model and ESIC Hospitals and Super Specialty hospitals. As far as ESIC contribution is concerned we have taken together the last two heads and distributed that into the categories of employee and employer contribution using the predefined ration of 1.75 and 4.75 respectively.

***Railway***

Two different secondary sources of data namely; the demands for grants (DDGs) volume-2 for expenditure of the central government on railways for the year 2015 and the website of health directorate of Indian Railway^^[[2]](#footnote-2)^^ has been used to calculate health expenditure by Railway department. The DDG budget provides information on health expenditure by different heads under three different demand numbers (i.e. 11, 12 and 13). Minor heads like Medical services (200), Medical, Health and Welfare (530) and Rastriya Swasthya Bima Yojana (660) have been culled out to arrive at total health expenditure. However, this data source does not provide information state-wise since the budget is prepared zone-wise. To arrive at state-wise health expenditure we used the information of bed strength of railway hospitals located in different state under different zone which is available in health directorate of Indian Railway website. For example, Northern Railway hospitals are located in different locations of Punjab. We segregated those hospitals by their place of location and accordingly added their bed strength. The state’s share of bed strength in their respective zone has been multiplied by respective zone’s total health expenditure to arrive at state health expenditure. Due to unavailability of state-wise railway employees data we have assumed that state-wise railway hospital bed strength represents the intensity of total number of staff employed and their healthcare needs. Numerically it can be defined as let’s say; Zone and State Health Expenditure is termed as ZHE and SHE respectively. Zone and state hospital bed strength is represented by the term ZHB and SHB respectively. Then, state health expenditure is calculated as

$$SHE= \delta* ZHE$$

Where, $\delta= SHB/ZHB$

***Out of Pocket Expenditures (OOPE)***

We have followed per capita method to estimate the OOPE from NSS and Census data –

1. Population projection

The last information on Census is available for 2011. However, we have attempted to estimate the State Health Accounts for 2013-14 and the NSS data has been collected during January – June 2014. So, we have projected the population for the 2014; using the census population from 2001 and 2011 data. The annual growth rate of population has been estimated using the formula –

$$\alpha=[(\frac{P_{2011}}{P_{2001}})^{\frac{1}{10}}-1]X100$$

Where,

α = annual growth rate of population;

P_2011_ = Population in 2011;

P_2001_ = population in 2001;

Using the annual growth rate of population (α), we have projected the population for 2014 by using the formula –

$P_{2014}=P_{2011}(1+\frac{\alpha}{100})^{(2014-2011)}$

Where P_2014_ = population in 2014;

1. Per-capita OOPE Estimation

Using the NSS unit level data we have estimated the total OOP expenditure for each component (package, consultancy fee, bed charge, medicine etc.) of in-patient care, out-patient care and child birth. NSS also provides information on reimbursement from different sources (like medical insurance company or employer) for these three services. We have deducted the reimburse amount from each component of the medical care facilities for IP, OP and child birth^[[3]](#footnote-3)^ to get the net OOP expenditure for each component at the individual level. The net OOP expenditure of all individuals (who are utilizing the services)for each component has been added to estimate the total OOP expenditure for each component (like total OOP expenditure for package in IP, total OOP expenditure for medicine in OP etc.) in the state. NSS provides information on each episode of illness and the facilities (formal or informal) utilized for treatment. However, NSS does not provide separate information for expenditure on formal and informal care, if a person utilized both type of care for treatment (for multiple visit). We have applied separate methodology (see Annexure-A for details) to estimate the OOP expenditure for each episode separately for these multiple visits. NSS only provides the total expenditure (no separate information is available for different components) for ANC and PNC care during the 365 days reference period. We have used this information to estimate the total OOP expenditure for ANC and PNC care separately. The total expenditure for each component has been divided with the total NSS (projected) population to generate the per-capita OOP expenditure for each service component of IP, OP, child birth and the per capita OOP expenditure for ANC and PNC. As the information of out-patient care has been provided for 15 days reference period, we have annualized the total OOP expenditure by multiplying it by 24.33 (365/15).

1. Total OOPE estimation

Now we have multiplied the per capita OOPE with the projected 2014 population to get the total OOPE for those services components and ANC and PNC.

**Expenditure on Vaccination**

Pharmatrac data has been used to estimate the OOPE on vaccination in Punjab. It provides data for some of the Indian states and union territories including Punjab. We have used the population share for these states & UTs to estimate the state/UT wise expenditure on vaccination.

**Voluntary Health Insurance (VHI)**

From NSS 2014 data, we have estimated the utilization of VHI share for each state/UT and the average premium paid for the states/UTs. We have multiplied the estimated population of 2014 with the utilization share of the VHI to get the total VHI utilization during 2014 of a particular state. Then we have multiplied the average premium paid for health insurance with the total utilization to get the total VHI amount for the state. However, the estimated total VHI amount was very low compared to the VHI amount provided by the Insurance Regulatory and Development Authority (IRDA) at the all India level (2013-14). So, we have weighted the total VHI amount available from IRDA by the corresponding state’s share in total VHI (as estimated from NSS data for 2014) to get state wise actual OOP expenditure on VHI.

**Medical devices & Family Planning Expenditure**

We have estimated the per capita annual OOP expenditure on medical devices and family planning from NSS consumer expenditure survey (2011-12). We have inflated the expenditure amount from 2011-12 to 2014^[[4]](#footnote-4)^. Then we have multiplied the OOPE amount with the total projected population of 2014 to get the total OOPE amount on medical devices and family planning.

***Firms***

Using the data collected in our survey, expenditure on health benefits per employee has been estimated. Keeping in mind the requirements of system of health accounts, these estimated figures have been further broken up into different ways through which this expenditure was made. In case of expenditure on corporate social responsibility and enterprises’ own facilities, per unit expenditure has been estimated.

Fifth economic census has been used as first step of blow up of sample estimates for private enterprises. Public sector units, non-profit institutions and unincorporated units have been left out from the universe of units listed in fifth economic census. This exercise has been carried out for primary, secondary and tertiary sectors separately. Employment size from the census and sample estimates of expenditure per employee have been used to arrive at total expenditure on employees’ health benefits for these three sectors separately. Employment figures from Department of Public Sector Enterprises (DPE), Government of India and state audited reports from Comptroller General Audit, Government of India have been used in case of public sector units.

Sample estimate of per unit health expenditure and number of units obtained from same sources as in case of expenditure on health benefits, have been used to blow up the expenditure on corporate social responsibility and enterprises’ own facilities.

Since fifth economic census is quite outdated, figures of employment and number of units have been adjusted using provisional report of sixth economic census.

***Non-Governmental Organizations***

The ‘blow-up strategy’ from sample statistics (sample mean and ratio) is intended to use latest (*sixth*) Economic Census [[2](#_ENREF_2)] information to arrive at state/national level estimates. But, given the fact that unit level information of *sixth* Economic Census are not available in public domain as of date, the CSO-NAD NPIs Census information may provisionally be used. The weighting patterns for aggregation as well as ratio estimate at state level are designed separately for respective groups of NGOs^^[[5]](#footnote-5)^^.

**Apportioning Statistics**

The approach followed for constructing health accounts is generally top down in nature, where the all the expenses are captured on accrual basis. Top down approach generally provide the data on expenditures comprised of transactions done in a period. Now, as the same set of resources are consumed for provisioning of healthcare services generally delivered as an integrated package i.e. curative care, preventive care, promotive care etc. and therefore, difficult to allocate the resources consumed for particular type of service. Besides, dealing with difficulty in allocating resources consumed for different service provisions by their nature (curative care, preventive care, promotive care etc.), apportioning of resources consumed for out-patient care, in-patient care and day care etc. which falls within the domain of curative care, introduces a second set of complexity. So, the apportioning of expenditures by functional classification was the major challenge faced while constructing SNHA for Punjab.

Primary Health Centres (PHCs) and Community Health Centres (CHCs)

Using the information from costing studies mentioned above particularly for PHCs and CHCs of Punjab, the apportioning statistics was designed. At the level of PHCs and CHCs, there is provision of both preventive and curative care services and therefore, annual cost of resources consumed at the level of health facilities were disaggregated. The ratio of resources consumed for providing health care services to a patient through OPD: IPD department was assumed to be 1:4.96 and 1:3.98 for PHC and CHC respectively, using the unit cost estimates from literature [[3](#_ENREF_3)] (Suppl. Appendix Table S1).

District Hospital (DH)

At the level of district hospitals, where the service provisioning is largely of curative nature, the expenditures in a financial year were apportioned into three main categories i.e. outpatient care, inpatient care and day care services corresponding to the functional classification given by SHA 2011. A day care procedure is generally described as patient treatment which requires stay at health facility but less than 24 hours like for a cataract surgery, dialysis procedure etc. Minor surgical procedures conducted at sampled health facilities in the study period of costing studies were considered the proxy of day care services where the patient stay at the health facility was less than 24 hours. After apportion the day care part, remaining part was apportioned for outpatient and inpatient curative services. The ratio of resources consumed for providing health care service to a patient through OPD: IPD department was assumed to be 1:4.97 using the unit cost [[4](#_ENREF_4)] (Suppl. Appendix Table S2).

Tertiary Care and Teaching Hospital (PGIMER)

A tertiary care hospital and teaching institute (PGIMER) located at Chandigarh (U.T), caters the population of five states including Punjab for providing specialized healthcare services. As per the hospital statistics by the regional distribution of beneficiaries, Punjab had a share of almost 30% in the total patient load. Therefore, the grants given by Central Government to run this institute were also taken into consideration while preparing the SNHA for Punjab. Firstly, the overall expenditure of the institute in 2013-14 was apportioned into two main classifications i.e. teaching and patient care. Some part of the overall grant received for the institute is specifically given in the name of ‘Education and Training’. Besides this, the salaries/compensations given to the institute faculty, students and administrative staff was also apportioned who are involved directly or indirectly in teaching and training programme based on their level of effort (LOE) towards it.

For LOE estimation, few departments were selected randomly i.e. internal medicine, paediatrics, orthopaedics, community medicine, medical microbiology, medical parasitology, biophysics, biochemistry, histopathology, experimental medicine and bio-technology and physiotherapy and their teaching rosters, schedules of journal clubs, seminars and other teaching/training activities for next 6 months were obtained. Besides the classroom teaching, the time spent on clinical teaching rounds in the inpatient wards and case discussions in outpatient departments were also considered by conducting interviews with people involved in it. Based on these teaching roasters and interviews, the time spent by faculties on teaching out of their total working hours was estimated for the reference period. While doing these computations, the teaching activities were divided into two parts i.e. activities with role of a single faculty (classroom teachings, clinical rounds, case discussion etc.) and activities with participation of all faculties (journal clubs, seminars etc.). For students different approach was used because to participate in these activities is a compulsion for students but we assumed 75% attendance of students for each activity. The estimated average time (out of total working hours) which goes for teaching in the institute was 18.5%. This percentage was applied on salaries of faculties, students and administrative staff which are 25% of overall workforce in the institute but constitutes 63% of overall salary expenditure. This apportioned share of teaching was 10.7% of overall salary expenditure and is used as final apportioning factor for teaching.

Secondly, the expenditure share for patient care was further disaggregated into three main classifications which is outpatient care, inpatient care and day care. As in case of DH, minor surgeries were taken as proxy of day care procedures which also includes procedures like dialysis, chemotherapy, blood transfusion etc. in a tertiary care setting. The ratio of resources consumed for providing health care service to a patient on OPD: IPD: Day care was assumed to be 1:10:2 using the unit cost estimates from literature [[5](#_ENREF_5)]. Finally, all the disaggregated expenditures were apportioned for Punjab which was 30% of total expenditures based on its share in terms of patient load (Suppl. Appendix Table S3).

**Table A: Apportioning keys for Primary Health Centre (PHC) and Community Health Centre (CHC)**

| **Parameters** | **Formulae** | **PHC** | **CHC** |
| --- | --- | --- | --- |
| Total estimated cost of resources consumed at sampled PHCs in Punjab in a financial year | X |  |  |
| Proportion share of preventive services | a | 0.209 | 0.116 |
| Proportion share of curative services | 1-a | 0.791 | 0.885 |
| Patients treated in outpatient care department | y | 25958 | 64661 |
| Patients treated in inpatient care department | z | 387 | 3195 |
| Total patients treated (Out-patient + Inpatient care) | y+z | 26345 | 67856 |
| Ratio of resources consumed for per case treated OPD:IPD | m:n | (1):(4.968) | (1): (3.988) |
| Share of patient load- outpatient department | y/(y+z) | 0.985 | 0.953 |
| Share of patient load- inpatient department | z/(y+z) | 0.015 | 0.047 |
| Average length of stay (ALOS)- IPD (e ) | e | 1.98 | 1.99 |
| OPD share * Ratio of resources consumed | Y= (y/(y+z))*m | 0.985 | 0.953 |
| IPD share * Ratio of resources consumed * ALOS | Z=(z/(y+z))*n*e | 0.144 | 0.374 |
| Factor sum | Y+Z | 1.130 | 1.327 |
| Final OPD apportioning factor | (Y/(Y+Z))*(1-a) | 0.690 | 0.635 |
| Final IPD apportioning factor | (Z/(Y+Z))*(1-a) | 0.101 | 0.249 |

**Table B: Apportioning keys for District Hospital (DH)**

| **Parameters** | **Formulae** | **DH** |
| --- | --- | --- |
| Proportion share of day care services | a | 0.1 |
| Proportion share of outpatient and inpatient curative services | 1-a | 0.9 |
| Patients treated in outpatient care department | y | 575392 |
| Patients treated in inpatient care department | z | 26509 |
| Total patients treated (Out-patient + Inpatient care) | y+z | 601901 |
| Ratio of resources consumed for per case treated OPD:IPD | m:n | (1):(4.97) |
| Share of patient load- outpatient department | y/(y+z) | 0.956 |
| Share of patient load- inpatient department | z/(y+z) | 0.044 |
| Average length of stay (ALOS)- IPD (e ) | e | 2.5 |
| OPD share * Ratio of resources consumed | Y= (y/(y+z))*m | 0.956 |
| IPD share * Ratio of resources consumed * ALOS | Z=(z/(y+z))*n*e | 0.547 |
| Factor sum | Y+Z | 1.503 |
| Final OPD apportioning factor | (Y/(Y+Z))*b | 0.572 |
| Final IPD apportioning factor | (Z/(Y+Z))*b | 0.328 |

**Table C: Apportioning keys for tertiary care hospital, PGIMER**

| **Parameters** | **Formulae** | **PGIMER** |
| --- | --- | --- |
| Proportion share of day care services | a | 0.067 |
| Proportion share of outpatient and inpatient curative services | 1-a | 0.933 |
| Patients treated on day care basis | x | 144078 |
| Patients treated in outpatient care department | y | 1917833 |
| Patients treated in inpatient care department | z | 78568 |
| Total patients treated (Outpatinet + Inpatient care) | x+y+z | 2140479 |
| Ratio of resources consumed for per case treated Day care:OPD:IPD | l:m:n | (2):(1):(10) |
| Share of patient load- day care | x/(x+y+z) | 0.067 |
| Share of patient load- outpatient department | y/(x+y+z) | 0.896 |
| Share of patient load- inpatient department | z/(x+y+z) | 0.037 |
| Average length of stay (ALOS)- IPD (e ) | e | 4.48 |
| Day care share * Ratio of resources consumed | X= (x/(x+y+z)*l | 0.135 |
| OPD share * Ratio of resources consumed | Y= (y/(y+z))*m | 0.896 |
| IPD share * Ratio of resources consumed * ALOS | Z=(z/(y+z))*n*e | 1.644 |
| Factor sum | X+Y+Z | 2.675 |
| Final Day care apportioning factor | (Y/(X+Y+Z)) | 0.050 |
| Final OPD apportioning factor | (Y/(X+Y+Z))*(1-a) | 0.313 |
| Final IPD apportioning factor | (Z/(X+Y+Z))*(1-a) | 0.574 |

**Results**

**Table D: Overall health expenditure by financing schemes in Punjab, 2013-14**

| **Financing scheme** | **Name of Categories** | **Expenditure (INR Crores)** | **Share (%)** | **Per capita (INR)** |
| --- | --- | --- | --- | --- |
|  |  |  |  |  |
| Government scheme and compulsory Heath care FS | Central Sector Scheme | 146.3 | 1.09 | 52.8 |
|  | Centre Sponsored Scheme | 390.8 | 2.90 | 140.9 |
|  | State Department of Health | 1813.4 | 13.46 | 653.7 |
|  | Other departments | 186.5 | 1.38 | 67.2 |
|  | Local Bodies | 66.2 | 0.49 | 23.8 |
|  | Tax Financed Insurance Scheme | 2.2 | 0.02 | 0.8 |
|  | Social Health Insurance Schemes | 274.5 | 2.04 | 98.9 |
|  | Total | 2879.8 | 21.38 | 1038.2 |
| Voluntary Health care payment scheme | NPISH Financing Schemes (NGOs) | 55.9 | 0.42 | 20.2 |
|  | Enterprises (private firms except health care providers) financing schemes | 261.6 | 1.94 | 94.3 |
|  | Total | 317.5 | 2.36 | 114.5 |
| Household –out-of –pocket payment | Out-of-pocket excluding cost-sharing | 10244.9 | 76.07 | 3693.2 |
|  | Cost sharing with voluntary insurance schemes | 25.4 | 0.19 | 9.2 |
|  |  | 10270.3 | 76.26 | 3702.4 |
|  | Overall | 13467.7 | 100 | 4855 |

**Table E: Classification of total health expenditure based on healthcare agents, 2013-14**

| **Name of Categories** | **Expenditure**  **(INR Crores)** | **Share (%)** |
| --- | --- | --- |
| Central Government | 254.0 | 1.9 |
| State Government | 1866.2 | 13.9 |
| State Health Society | 230.4 | 1.7 |
| Local bodies | 66.2 | 0.5 |
| Social Security Agency | 274.5 | 2.0 |
| All other general govt. units | 186.5 | 1.4 |
| Govt. Insurance Agency | 2.2 | 0.0 |
| Corporations/firms (Other than providers of health services) | 261.6 | 1.9 |
| Non-profit institutions serving households (NGOs) | 55.9 | 0.4 |
| Households | 10270.3 | 76.3 |
| ALL Financing Agents | 13467.7 | 100.0 |

**Table F: Classification of total health expenditure based on healthcare functions, 2013-14**

| **Function** | **Name of categories** | **Expenditure**  **(Rs Crore)** | **Share (%)** |
| --- | --- | --- | --- |
| Curative care | Inpatient curative care | 2696.4 | 20.02 |
|  | Day curative care | 428.5 | 3.18 |
|  | Outpatient curative care | 1766.0 | 13.11 |
|  | Home-based curative care | 35.2 | 0.26 |
|  | Total | 4926.1 | 36.58 |
| Rehabilitative Care | Outpatient rehabilitative care | 0.1 | 0.001 |
| Ancillary services (non-specified by function) | Laboratory services | 548.2 | 4.07 |
|  | Imaging services | 538.6 | 4.00 |
|  | Patient transportation | 425.4 | 3.16 |
|  | Total | 1512.3 | 11.23 |
| Medical goods (non-specified by function) | Pharmaceuticals and other medical non-durable goods | 5602.3 | 41.60 |
|  | Therapeutic appliances and other medical goods | 3.1 | 0.02 |
|  | Total | 5605.4 | 41.62 |
| Preventive care | Information, education and counselling programmes | 405.2 | 3.01 |
|  | Immunisation programmes | 130.0 | 0.97 |
|  | Early disease detection programmes | 139.9 | 1.04 |
|  | Healthy condition monitoring programmes | 390.4 | 2.90 |
|  | Epidemiological surveillance and risk and disease control programmes | 47.1 | 0.35 |
|  | Preparing for disaster and emergency response programmes | 0.2 | 0.001 |
|  | Total | 1112.9 | 8.26 |
| Governance, and health system and financing administration | Governance and Health system administration | 234.6 | 1.74 |
|  | Administration of health financing | 73.7 | 0.55 |
|  | Total | 308.2 | 2.29 |
| Other health care services not elsewhere classified (n.e.c.) | Other health care services not elsewhere classified (n.e.c.) | 2.8 | 0.02 |
|  | **Overall** | 13467.7 | 100.00 |

**Table G: Total health expenditure by Providers in Punjab, 2013-14**

| **Provider** | **Codes** | **Name of categories** | **Expenditure**  **(INR Crores)** | **Share (%)** |
| --- | --- | --- | --- | --- |
| Hospitals | HP.1.1 | General hospitals | 3632.0 | 26.97 |
|  | HP.1.2 | Mental health hospitals | 10.5 | 0.08 |
|  | HP.1.3 | Specialized hospitals (other than mental health hospitals) | 331.4 | 2.46 |
|  | Total |  | 3973.9 | 29.51 |
| Residential Long-term care facilities | HP.2.1 | Long Term nursing care facilities | 0.1 | 0.001 |
| Providers of ambulatory health care | HP.3.1 | Medical practices | 813.7 | 6.04 |
|  | HP.3.3 | Other health care practitioners | 94.8 | 0.70 |
|  | HP.3.4 | Ambulatory health care centres | 590.9 | 4.39 |
|  | Total |  | 1499.3 | 11.13 |
| Providers of ancillary services | HP.4.1 | Providers of patient transportation and emergency rescue | 392.2 | 2.91 |
|  | HP.4.2 | Medical and diagnostic laboratories | 1079.3 | 8.01 |
|  | HP.4.9 | Other providers of ancillary services | 39.3 | 0.29 |
|  | Total |  | 1510.8 | 11.22 |
| Retailers and other providers of medical goods | HP.5.1 | Pharmacies | 5564.4 | 41.32 |
|  | HP.5.2 | Retail sellers and other suppliers of durable medical goods and medical appliance | 2.4 | 0.02 |
|  | Total |  | 5566.8 | 41.33 |
| Providers of preventive care | HP.6 | Providers of preventive care | 456.8 | 3.39 |
| Providers of health care system administration and financing | HP.7.1 | Government health administration agencies | 264.1 | 1.96 |
|  | HP.7.2 | Social health insurance agencies | 170.5 | 1.27 |
|  | HP.7.3 | Private health insurance administration agencies | 22.6 | 0.17 |
|  | Total |  | 457.2 | 3.39 |
|  | HP.9 | Rest of the world | 2.8 | 0.02 |
|  | Total |  | 13467.7 | 100.00 |

**Table H: Factors of provision for the state of Punjab in 2013-14**

| **Factors** | **Name of categories** | **Expenditure**  **(in Lacs)** | **Percent** |
| --- | --- | --- | --- |
| Compensation of employees | Wages and Salary | 2783 | 22.50 |
|  | All other Costs related to employees | 71 | 0.57 |
| Self-Employed professional Remuneration | Self-employed Professional Remuneration | 29.8 | 0.24 |
| Material and Services used | Health Services | 3378.2 | 27.32 |
|  | Health Goods | 5664 | 45.80 |
|  | Non Health Services | 393 | 3.18 |
|  | Non Health Goods | 22 | 0.18 |
| Other items of spending on inputs | Other items of spending | 25.4 | 0.21 |
|  | All factors of provision | 12366.9 | 100.00 |

**Table I: Financial Flows by Financing scheme (HF) and Function (HC), Punjab-2013-14 (in crores)**

| Description | Central Sector Scheme | Centre Sponsored Scheme | State Department of Health | Other departments | Urban Local Bodies | Tax- Financed Insurance Scheme | Social Health Insurance Schemes | NPISH Financing Schemes (Exluding HF 2.2.2) | Enterprises (except health care providers) financing schemes | Out-of-pocket excluding cost-sharing | Cost- sharing with voluntary insurance schemes | All |
| --- | --- | --- | --- | --- | --- | --- | --- | --- | --- | --- | --- | --- |
| Inpatient Curative Care | 34.2 | 28.5 | 292.4 | 0.0 | 0.0 | 2.0 | 115.3 | 1.5 | 67.7 | 2111.4 | 25.4 | 2678.3 |
| Day Curative Care | 12.1 | 0.3 | 74.2 | 0.0 | 0.0 | 0.2 | 27.4 | 2.2 | 20.5 | 0.0 | 0.0 | 137.0 |
| Outpatient Curative Care | 80.8 | 6.6 | 773.6 | 186.5 | 60.4 | 0.0 | 131.7 | 14.3 | 151.0 | 747.0 | 0.0 | 2152.1 |
| Home-Based Curative Care | 0.0 | 0.4 | 0.2 | 0.0 | 0.0 | 0.0 | 0.0 | 0.0 | 0.1 | 33.1 | 0.0 | 33.8 |
| Outpatient Rehabilitative Care | 0.0 | 0.0 | 0.0 | 0.0 | 0.0 | 0.0 | 0.0 | 0.1 | 0.0 | 0.0 | 0.0 | 0.1 |
| Laboratory Services | 0.3 | 4.0 | 6.6 | 0.0 | 0.0 | 0.0 | 0.0 | 1.4 | 0.0 | 75.0 | 0.0 | 87.3 |
| Imaging Services | 1.4 | 0.0 | 0.0 | 0.0 | 0.0 | 0.0 | 0.0 | 1.3 | 0.0 | 996.8 | 0.0 | 999.5 |
| Patient Transportation | 0.0 | 0.4 | 39.5 | 0.0 | 0.0 | 0.0 | 0.0 | 1.3 | 0.0 | 384.3 | 0.0 | 425.4 |
| Pharmaceuticals and other medical non-durable goods | 13.3 | 35.0 | 19.4 | 0.0 | 2.4 | 0.0 | 0.0 | 0.0 | 0.0 | 5532.2 | 0.0 | 5602.3 |
| Therapeutic Appliances and other medical goods | 1.6 | 1.4 | 0.0 | 0.0 | 0.0 | 0.0 | 0.0 | 0.0 | 0.0 | 0.1 | 0.0 | 3.1 |
| Information, Education and Counselling Programmes (IEC) | 0.0 | 176.6 | 183.5 | 0.0 | 0.0 | 0.0 | 0.0 | 13.8 | 2.8 | 0.0 | 0.0 | 376.7 |
| Immunisation programmes | 0.0 | 11.4 | 13.6 | 0.0 | 0.0 | 0.0 | 0.0 | 3.4 | 6.5 | 64.0 | 0.0 | 98.8 |
| Early Disease Detection Programmes | 0.0 | 0.4 | 128.8 | 0.0 | 0.0 | 0.0 | 0.0 | 10.7 | 0.0 | 0.0 | 0.0 | 139.9 |
| Healthy Condition Monitoring Programmes | 0.0 | 23.2 | 42.8 | 0.0 | 0.0 | 0.0 | 0.0 | 1.6 | 6.5 | 301.0 | 0.0 | 375.0 |
| Epidemiological Surveillance and Risk and Disease Control Programmes | 0.0 | 32.0 | 5.3 | 0.0 | 3.3 | 0.0 | 0.0 | 0.0 | 6.5 | 0.0 | 0.0 | 47.1 |
| Preparing for disaster and emergency response programmes | 0.0 | 0.2 | 0.0 | 0.0 | 0.0 | 0.0 | 0.0 | 0.0 | 0.0 | 0.0 | 0.0 | 0.2 |
| Governance and Health System Administration | 2.6 | 69.1 | 162.8 | 0.0 | 0.0 | 0.0 | 0.0 | 0.0 | 0.0 | 0.0 | 0.0 | 234.6 |
| Administration of Health Financing | 0.0 | 0.0 | 69.3 | 0.0 | 0.0 | 0.0 | 0.0 | 4.3 | 0.0 | 0.0 | 0.0 | 73.7 |
| Other health care Services not elsewhere classified (n.e.c.) | 0.0 | 1.3 | 1.4 | 0.0 | 0.0 | 0.0 | 0.0 | 0.0 | 0.0 | 0.0 | 0.0 | 2.8 |
| All above ie Total of above? | 146.3 | 390.8 | 1813.4 | 186.5 | 66.2 | 2.2 | 274.5 | 55.9 | 261.6 | 10244.9 | 25.4 | 13467.7 |

**Table J: Financial Flows by revenue from Financing Schemes (HF) and Financing agent (FA), Punjab-2013-14 (in crores)**

| Description | Central Sector Scheme | Centre Sponsored Scheme | State Department of Health | Other departments | Urban Local Bodies | Tax Financed Insurance Scheme | Social Health Insurance Schemes | NPISH Financing Schemes (Exluding HF 2.2.2) | Enterprises (except health care providers) financing schemes | Out-of-pocket excluding cost-sharing | Cost- sharing with voluntary insurance schemes | ALL the above |
| --- | --- | --- | --- | --- | --- | --- | --- | --- | --- | --- | --- | --- |
| Central Government | 146.3 | 107.6 | 0.0 | 0.0 | 0.0 | 0.0 | 0.0 | 0.0 | 0.0 | 0.0 | 0.0 | 254.0 |
| State Government | 0.0 | 123.3 | 1742.8 | 0.0 | 0.0 | 0.0 | 0.0 | 0.0 | 0.0 | 0.0 | 0.0 | 1866.2 |
| State Health Society | 0.0 | 159.9 | 70.6 | 0.0 | 0.0 | 0.0 | 0.0 | 0.0 | 0.0 | 0.0 | 0.0 | 230.4 |
| Local bodies | 0.0 | 0.0 | 0.0 | 0.0 | 66.2 | 0.0 | 0.0 | 0.0 | 0.0 | 0.0 | 0.0 | 66.2 |
| Social Security Agency | 0.0 | 0.0 | 0.0 | 0.0 | 0.0 | 0.0 | 274.5 | 0.0 | 0.0 | 0.0 | 0.0 | 274.5 |
| All other general govt. units | 0.0 | 0.0 | 0.0 | 186.5 | 0.0 | 0.0 | 0.0 | 0.0 | 0.0 | 0.0 | 0.0 | 186.5 |
| Govt. Insurance Agency | 0.0 | 0.0 | 0.0 | 0.0 | 0.0 | 2.2 | 0.0 | 0.0 | 0.0 | 0.0 | 0.0 | 2.2 |
| Corporations (Other than providers of health services) | 0.0 | 0.0 | 0.0 | 0.0 | 0.0 | 0.0 | 0.0 | 0.0 | 261.6 | 0.0 | 0.0 | 261.6 |
| Non-profit institutions serving households (NPISHs) | 0.0 | 0.0 | 0.0 | 0.0 | 0.0 | 0.0 | 0.0 | 55.9 | 0.0 | 0.0 | 0.0 | 55.9 |
| Households | 0.0 | 0.0 | 0.0 | 0.0 | 0.0 | 0.0 | 0.0 | 0.0 | 0.0 | 10244.9 | 25.4 | 10270.3 |
| ALL Financing Agents | 146.3 | 390.8 | 1813.4 | 186.5 | 66.2 | 2.2 | 274.5 | 55.9 | 261.6 | 10244.9 | 25.4 | 13467.7 |

**Table K: Financial Flows by Financing scheme (HF) and Provider (HP), Punjab-2013-14 (in crores)**

| Description | Central Sector Scheme | Centre- Sponsored Scheme | State Department of Health | Other departments | Urban Local Bodies | Tax- Financed Insurance Scheme | Social Health Insurance Schemes | NPISH Financing Schemes (Excluding HF 2.2.2) | Enterprises (except healthcare providers) financing schemes | Out-of-pocket excluding cost-sharing | Cost- sharing with voluntary insurance schemes | ALL the above |
| --- | --- | --- | --- | --- | --- | --- | --- | --- | --- | --- | --- | --- |
| General Hospitals | 0.0 | 35.8 | 738.9 | 186.5 | 60.4 | 2.2 | 274.5 | 0.9 | 208.7 | 2111.4 | 25.4 | 3644.7 |
| Mental Health Hospitals | 0.0 | 0.0 | 10.5 | 0.0 | 0.0 | 0.0 | 0.0 | 0.0 | 0.0 | 0.0 | 0.0 | 10.5 |
| Specialised Hospitals | 146.0 | 0.0 | 184.2 | 0.0 | 0.0 | 0.0 | 0.0 | 0.0 | 1.1 | 0.0 | 0.0 | 331.4 |
| Long-term nursing care facilities | 0.0 | 0.0 | 0.0 | 0.0 | 0.0 | 0.0 | 0.0 | 0.1 | 0.0 | 0.0 | 0.0 | 0.1 |
| Medical Practices | 0.0 | 0.0 | 0.0 | 0.0 | 0.0 | 0.0 | 0.0 | 13.8 | 19.7 | 780.2 | 0.0 | 813.7 |
| Other healthcare practitioners | 0.0 | 0.0 | 94.8 | 0.0 | 0.0 | 0.0 | 0.0 | 0.0 | 0.0 | 0.0 | 0.0 | 94.8 |
| Ambulatory Healthcare Centres | 0.0 | 106.4 | 159.8 | 0.0 | 0.0 | 0.0 | 0.0 | 0.0 | 0.0 | 324.7 | 0.0 | 590.9 |
| Providers of patient transportation and emergency rescue | 0.3 | 4.4 | 1.9 | 0.0 | 0.0 | 0.0 | 0.0 | 1.3 | 0.0 | 384.3 | 0.0 | 392.2 |
| Medical and diagnostic laboratories | 0.0 | 0.0 | 4.8 | 0.0 | 0.0 | 0.0 | 0.0 | 2.7 | 0.0 | 1071.8 | 0.0 | 1079.3 |
| Other providers of ancillary services | 0.0 | 0.0 | 39.3 | 0.0 | 0.0 | 0.0 | 0.0 | 0.0 | 0.0 | 0.0 | 0.0 | 39.3 |
| Pharmacies | 0.0 | 35.0 | 19.4 | 0.0 | 1.5 | 0.0 | 0.0 | 0.0 | 0.0 | 5508.5 | 0.0 | 5564.4 |
| Retail Sellers and other suppliers of durable medical goods & medical appliances | 0.0 | 1.4 | 0.0 | 0.0 | 0.9 | 0.0 | 0.0 | 0.0 | 0.0 | 0.1 | 0.0 | 2.4 |
| Providers of Preventive Care | 0.0 | 137.4 | 198.2 | 0.0 | 3.3 | 0.0 | 0.0 | 31.8 | 22.2 | 64.0 | 0.0 | 456.8 |
| Government Health Administration Agencies | 0.0 | 69.1 | 190.7 | 0.0 | 0.0 | 0.0 | 0.0 | 4.2 | 0.0 | 0.0 | 0.0 | 264.1 |
| Social Health Insurance Agencies | 0.0 | 0.0 | 169.4 | 0.0 | 0.0 | 0.0 | 0.0 | 1.1 | 0.0 | 0.0 | 0.0 | 170.5 |
| Private Health Insurance Administration Agencies | 0.0 | 0.0 | 0.0 | 0.0 | 0.0 | 0.0 | 0.0 | 0.0 | 9.9 | 0.0 | 0.0 | 9.9 |
| Rest of World | 0.0 | 1.3 | 1.4 | 0.0 | 0.0 | 0.0 | 0.0 | 0.0 | 0.0 | 0.0 | 0.0 | 2.8 |
| ALL the Above | 146.3 | 390.8 | 1813.4 | 186.5 | 66.2 | 2.2 | 274.5 | 55.9 | 261.6 | 10244.9 | 25.4 | 13467.7 |

**References**

1. ESIC. Annual Report: 2013-14. Employees' State Insurance Corporation. New Delhi. Available at: <http://www.esic.nic.in/publications.php>. 2014.

2. GoI. Sixth Economic Census. Central Statistics Office. Ministry of Statistics of Programme Implementation. Government of India. New Delhi2014.

3. Prinja S, Gupta A, Verma R, Bahuguna P, Kumar D, Kaur M, et al. Cost of Delivering Health Care Services in Public Sector Primary and Community Health Centres in North India. . PLoS ONE. 2016;11(8):e0160986. doi:10.1371/journal.pone.

4. Prinja S, Balasubramanian D, Jeet G, Verma R, Kumar D, Bahuguna P, et al. Cost of Delivering Secondary Level Health Care Services through Public Sector District Hospitals in India. Indian J Med Res (Forthcoming). 2017.

5. Chatterjee S, Levin C, Laxminarayan R. Unit Cost of Medical Services at Different Hospitals in India. PloS One. 2013;8(7):e69728.

1. There are **four** types of NGOs classified on the size of employment: Micro-NGO involving only volunteers with no formal employment, Small-NGO encompassing employment size up to 20, Medium-NGO ranging employment 20-100 and Large-NGO with employment more than 100. [↑](#footnote-ref-1)
2. <http://www.indianrailways.gov.in/railwayboard/uploads/directorate/health/health_1.jsp> accessed on 15 Jan 2016 [↑](#footnote-ref-2)
3. NSS classifies Package (only for IP and child birth), doctor’s fee, medicines, diagnostic test, bed charges (only for IP and child birth), other medical expenses and medicine-AYUSH (only for OP) as medical expenditure and Transport of patients and other non-medical expenditure as non-medical expenditure. Following SHA 2011, we have excluded other ‘non-medical expenditure’ (out of boundary) from our estimate. [↑](#footnote-ref-3)
4. Here we have taken the Consumer Price Index of industrial worker (CPI-IW) for the medical care only. [↑](#footnote-ref-4)
5. Evidently, a multi-stage stratified random sampling is designed for surveying the NGOs. In this complex sample design, though the proportional distribution is planned, aimed at the statewide analysis and stratum-specific minimum quota, the sample distribution seems to be differed from relative distribution of the population with respect to a variable in terms of both scale and proportion. In our stratified sampling process, suppose, population size is *N*, and allocated total sample size is *n* among *S* identified strata, the objective of weighting sample data is to confine its representativeness in relation to the study population. Applying weight(s) to sample seeks the goal of making sample largely like the population. In stratified sampling therefore an integrated weight may be defined as product of scale and proportional factors: $w_{s}=\frac{N}{n}\times\frac{{N_{s}}/N}{{n_{s}}/n}=\frac{N_{s}}{n_{s}}$, where the symbols are usual meaning. Seeing as our sampling realizes hierarchical scheme (multi-stage stratification), weights are in principle computed in the same way at each stage, and the final sapling fraction would be the product of probabilities in successive stages: $f_{sh}=\frac{n}{N}\times\frac{{n_{s}}/n}{{N_{s}}/N}\times\frac{{n_{sh}}/{n_{s}}}{{N_{sh}}/{N_{s}}}=\frac{n_{sh}}{N_{sh}}$, here *h* is a sub-stratum under the stratum *s*. Inverse of this sampling fraction is usually termed as base/design weight ($w_{sh}=\frac{N_{sh}}{n_{sh}})$. Subsequently, the base weight may be adjusted by two other factors: nonresponse error and sampling variance reduction at the post-survey period; our sample design is self-correcting to these factors. The statewide estimated weights to be applied with each unit level data are finally appeared in the following table. In this formulation, the population total at state level may be estimated in the following way:

   Suppose the total of a population variable $Y_{i}$ for a state s is denoted by $Y_{T}$. It is then estimated by the sample as $Y_{T}=\sum Y_{i}=\sum_{\boldsymbol{i\in}\boldsymbol{S}_{\boldsymbol{h}}} {\boldsymbol{w}_{\boldsymbol{shi}}\boldsymbol{y}}_{\boldsymbol{shi}}$ [↑](#footnote-ref-5)
